# Increasing floral visitation and hybrid seed production mediated by beauty mark in *Gossypium hirsutum*

**DOI:** 10.1111/pbi.13805

**Published:** 2022-03-22

**Authors:** Muhammad Ali Abid, Yunxiao Wei, Zhigang Meng, Yuan Wang, Yulu Ye, Yanan Wang, Haiyan He, Qi Zhou, Yanyan Li, Peilin Wang, Xianggan Li, Liuhua Yan, Waqas Malik, Sandui Guo, Chengcai Chu, Rui Zhang, Chengzhen Liang

**Affiliations:** ^1^ Biotechnology Research Institute Chinese Academy of Agricultural Sciences Beijing China; ^2^ Cropedit Biotechnology Co., Ltd Beijing China; ^3^ State Key Laboratory of Plant Genomics Institute of Genetics and Developmental Biology the Innovative Academy for Seed Design Chinese Academy of Sciences Beijing China; ^4^ College of Agriculture South China Agricultural University Guangzhou China

**Keywords:** *Gossypium hirsutum*, *Gossypium barbadense*, Beauty Mark, MYB, hybrid seed yield

## Abstract

Hybrid crop varieties have been repeatedly demonstrated to produce significantly higher yields than their parental lines; however, the low efficiency and high cost of hybrid seed production has limited the broad exploitation of heterosis for cotton production. One option for increasing the yield of hybrid seed is to improve pollination efficiency by insect pollinators. Here, we report the molecular cloning and characterization of a semidominant gene, *Beauty Mark* (*BM*), which controls purple spot formation at the base of flower petals in the cultivated tetraploid cotton species *Gossypium barbadense*. *BM* encodes an R2R3 MYB113 transcription factor, and we demonstrate that GbBM directly targets the promoter of four flavonoid biosynthesis genes to positively regulate petal spot development. Introgression of a *GbBM* allele into *G. hirsutum* by marker‐assisted selection restored petal spot formation, which significantly increased the frequency of honeybee visits in *G. hirsutum*. Moreover, field tests confirmed that cotton seed yield was significantly improved in a three‐line hybrid production system that incorporated the *GbBM* allele. Our study thus provides a basis for the potentially broad application of this gene in improving the long‐standing problem of low seed production in elite cotton hybrid lines.

## Introduction

Hybrid cotton varieties exhibit ~20% or higher yield increases over inbred varieties (Luo *et al*., 2013; Shahzad *et al*., 2019; Xing *et al*., 2017). However, the major technical limitation of hybrid cotton is the low pollination efficiency in hybrid seed production (Dai and Dong, 2014; Shahzad *et al*., 2019). Since cotton plants have indeterminate inflorescence with flowering time lasting for more than a month (Eaton, 1955; Huang *et al*., 2021), it is impossible to arrange for a single, large‐scale, and cross‐pollination event between two parents. This problem can be at least partially resolved through the improvement of pollination efficiency by insect pollinators (Moffet and Stith, 1972; Waller *et al*., 1985; Xing *et al*., 2005).

Cotton breeders have made considerable efforts to improve cross‐pollination efficiency by using honeybees as pollen carriers (Meena *et al*., 2003; Moffet and Stith, 1978; Vaissiere and Moffet, 1984), and although pollination efficiency can be enhanced by increasing the number of bees (Waller *et al*., 1985; Xia *et al*., 2019), the efficiency of honeybee‐mediated pollination remains low, and the yields of hybrid seed require substantial improvement before commercial deployment is feasible (Waller *et al*., 1985; Xia *et al*., 2019; Xing *et al*., 2005). It is well‐established that particular patterns of flower colors, such as petal spots, stripes, and blotches (caused by the deposition of pigments; Winkel‐Shirley, 2001; Zhang *et al*., 2014) can help to attract pollinators (Lunau, 2016; Sheehan *et al*., 2016) and thereby increase female reproductive success (Hopkins and Rausher, 2012). Accordingly, attempting to generate cotton varieties with flowers that are highly attractive to pollinators is a desirable genetic improvement strategy.

Allotetraploid cotton is a major global crop for natural fiber production (Paterson *et al*., 2012). *Gossypium hirsutum* and *Gossypium barbadense* comprise the vast proportion of cultivated species (Wang *et al*., 2019), and these differed substantially in both developmental timelines and physiological traits. *G. hirsutum* is widely planted around the world and accounts for more than 90% of the global cotton output (Huang *et al*., 2021; Li *et al*., 2015). *Gossypium barbadense* is characterized with a purple spot at the base of flower petals (Fryxell, 1984; Zhang *et al*., 2016) and petal spots enhance the attractiveness of *G. barbadense* flowers for insect pollinators, resulting in improved boll‐setting (Jones, 1996). Interestingly, the purple‐colored spot at the base of flower petals is a semidominant trait which is widespread in wild cotton species and often found in landraces of *G. hirsutum*, but was lost in most modern commercial *G. hirsutum* cultivars during selection (Zhang *et al*., 2016). Here, we show that the *Beauty Mark* (*GbBM*) gene from *G. barbadense*, a close allotetraploid relative of *G. hirsutum*, underlies activation of anthocyanin production, directly leading to formation of a pollinator‐attracting purple spot at the base of flower petals. Retrieval of beauty mark in *G. hirsutum* cultivars using marker‐assisted strategy can effectively increase pollinator visiting frequency and improve the three‐line system hybrid seed production in the main commercial cotton species *G. hirsutum*.

## Results

### Characterization of beauty mark phenotype in cotton population

**Figure 1:**
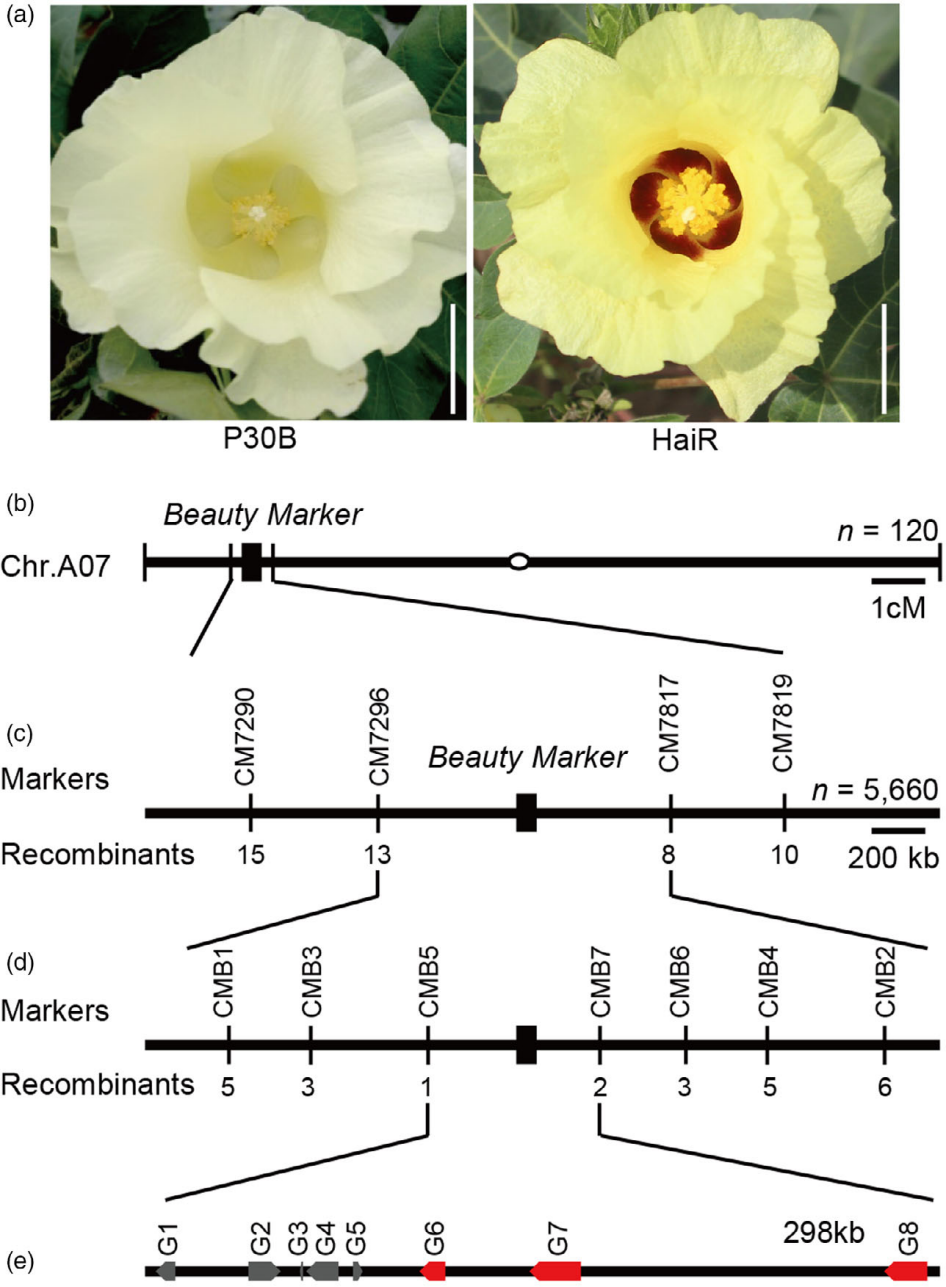
Cloning of the *GbBM* gene. (a) The flower “beauty mark” phenotypes of the P30B and HaiR lines. Scale bars, 1 cm. (b) Location of *GbBM* on *Gossypium barbadense* chromosome A07. (c) Coarse linkage map of *GbBM*. The number of recombinants in the F_2_ population derived by HaiR and P30B between the marker and *GbBM* is indicated. (d) High‐resolution linkage map of *GbBM*. (e) Annotation of the candidate region surrounding *GbBM* on *G. barbadense* chromosome A07 Arrows indicate putative genes the in the *G. barbadense* reference assembly. The red arrows represent three R2R3 MYB transcription factor genes.

To study the genetic basis underlying formation of the purple spot at the base of flower petals, we examined the prevalence of the purple spot phenotype across a wide range of tetraploid cotton varieties, including 312 *G. hirsutum* and 32 *G. barbadense* cultivars. All *G. barbadense* accessions carried the base purple spot on their flower petals; whereas 311 out of the 312 cultivated *G. hirsutum* genotypes were devoid of the petal spot. We therefore crossed spotted petal *G. barbadense* line HaiR with nonspotted petal *G. hirsutum* line P30B (Figure 1a). All of the interspecific F_1_ progeny had petal spots, which had an intermediate shade of color and size compared with HaiR (Figure S1). We then examined a large interspecific F_2_ offspring population comprising 5540 individuals. This population exhibited a phenotypic ratio of 3:1 (Table S1), suggesting that the purple spot at the petal base phenotype is a semidominant trait controlled by a single, semidominant locus. Since this purple spot at the base of flower petals resembled a purple beauty mark, we designated the underlying genetic locus in *G. barbadense* as *Beauty Mark* (*BM*).

### Map‐based cloning of *Beauty Mark* gene

To fine map the responsible gene in the *BM* locus responsible for this distinct phenotype, we initially developed 720 insertion‐deletion (InDel) markers which were distributed across the 26 cotton chromosomes using the published genome sequences of *G. barbadense* (Wang *et al*., 2019) and *G. hirsutum* (Li *et al*., 2015). Using 120 F_2_ plants that lacked the beauty mark phenotype, the *BM* locus was mapped to an interval between markers CM7296 and CM7817 on the short arm of chromosome A07 (Figure 1b–e and Figure S2). Furthermore, using 5660 F_2_ plants and newly developed InDel molecular markers (Table S2), we were able to narrow down the candidate gene to a ~298 kb genomic interval between markers CMB5 and CMB7 (Figure 1b–e). This region contains eight annotated genes (Table S3), including three tandem genes (*Gbar_A07G008290*, *Gbar_A07G008300*, and *Gbar_A07G008330*), which appear to be paralogue genes encoding putative R2R3 MYB transcription factors (TF) and sharing about 85% amino acid similarity.

**Figure 2:**
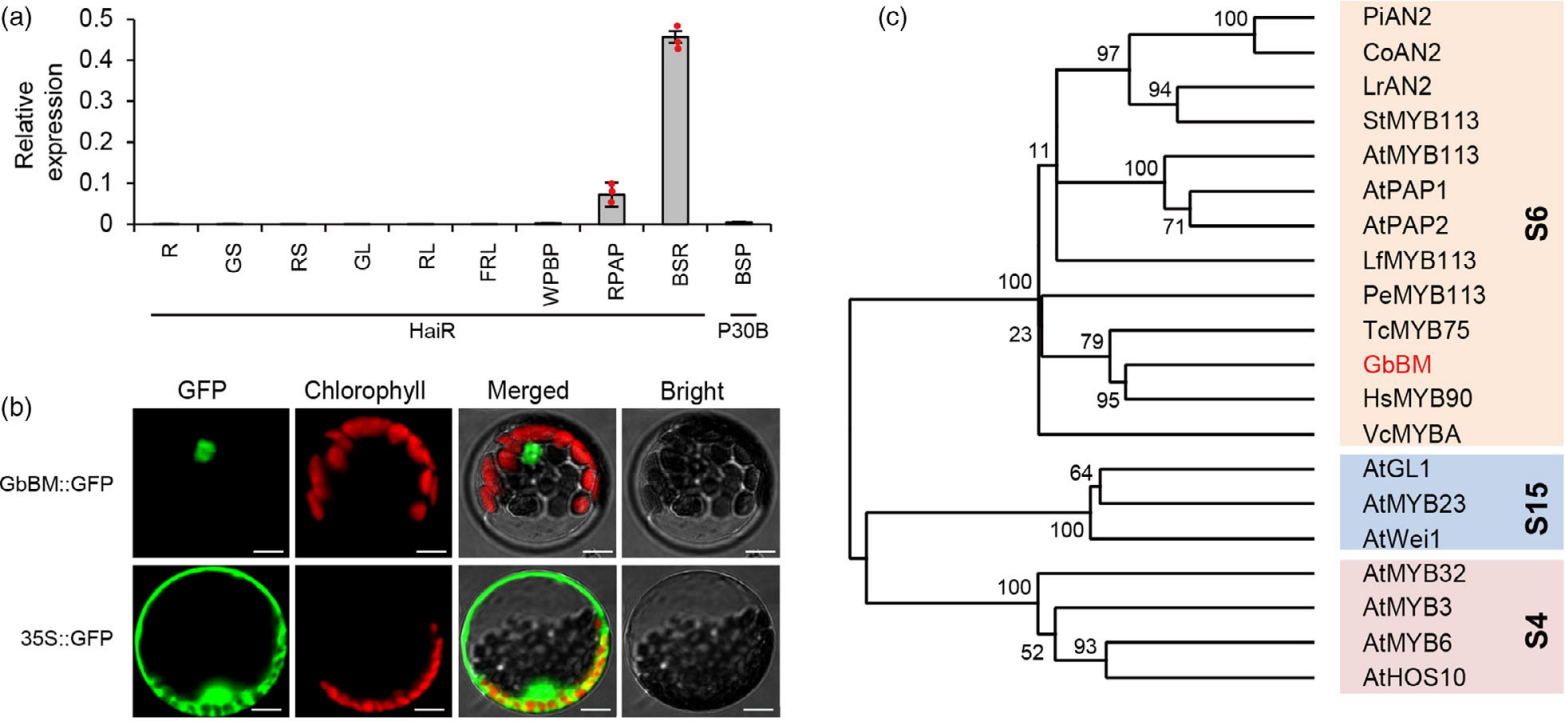
Expression pattern of *GbBM* gene and Phylogenetic analysis of the GbBM protein. (a) *GbBM* expression profile in various tissues. R, root; GS, green stem; RS, red stem; GL, green leaf; RL, red leaf under normal condition; FRL, frosted red leaf; WPBP, white petal before pollination; RPAP, red petal after pollination; BSR, bud spotted region of HaiR; BSP, bud‐spotted region of P30B. Bars are the mean SD of three biological replicates. (b) Subcellular localization of 35S::GbBM‐GFP (upper panel) and 35S::GFP (lower panel) in Arabidopsis protoplasts. Scale bars, 10 μm. (c) Phylogenetic analysis of GbBM (marked with red) and other MYBs using R2R3 MYB domains. Subgroup (S) names are indicated on the right, and the subgroups are colored.

Given that several R2R3 MYB TF have been implicated in pigment synthesis (Meng *et al*., 2019; Sheehan *et al*., 2016; Zhang *et al*., 2014), we first examined expression of these three candidate genes in both spotted and nonspotted regions of HaiR and P30A flower petals (Figure S3a–c). Only *Gbar_A07G008330* displayed differentially elevated expression in the beauty mark region of HaiR petals from the bud stage to the flowering stage compared with P30B. Further, the only detectable expression of *Gbar_A07G008330* occurred in red flower petals after pollination (Figure 2a), suggesting that mediates pigment formation in a tissue‐specific manner. *Gbar_A07G008330* encodes a nuclear‐localized protein belonging to the S6 clade of the R2R3 MYB TF subfamily (Figure 2b,c), which also includes the well‐characterized proteins PRODUCTION OF ANTHOCYANIN PIGMENT (AtPAP1 and AtPAP2) and ANTHOCYANIN2 (PiAN2, CoAN2, and LrAN2) (Dubos *et al*., 2010; Meng *et al*., 2019; Stracke *et al*., 2001). Thus, we focused on *Gbar_A07G008330* as the most promising candidate casual gene for the beauty mark phenotype.

**Figure 3:**
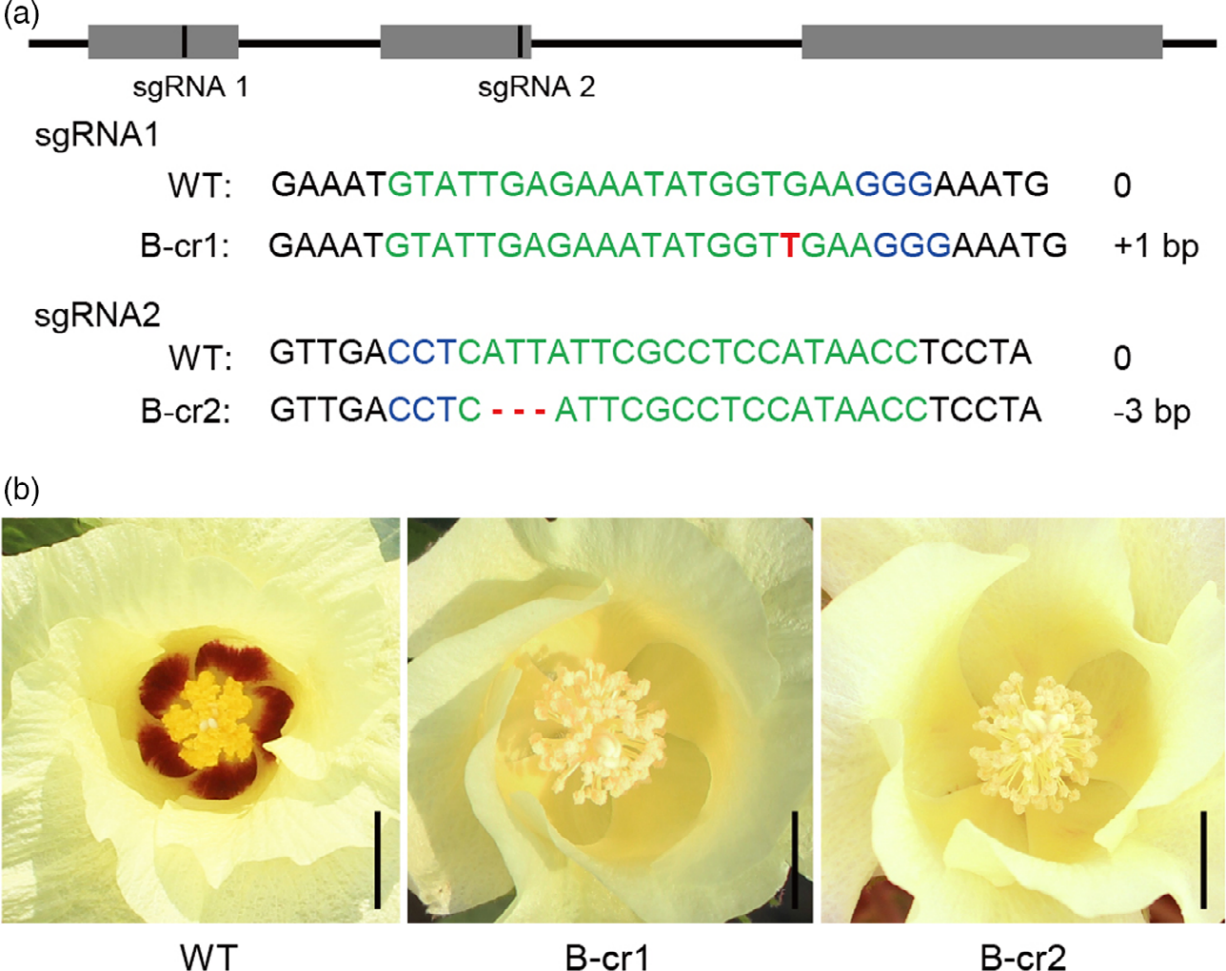
The phenotype of *GbBM* CRISPR/Cas9 lines. (a) Editing of the *GbBM* locus using CRISPR/Cas9 with two independent single‐guide RNAs (sgRNA1 for an inactivating insertion and sgRNA2 for an inactivating deletion). The sgRNA target sites and a protospacer‐adjacent motifs (PAM) are indicated in green and in blue, respectively. Insertions and deletions are indicated in red or by dashes, respectively. (b) Beauty mark flower phenotypes in wild type, B‐cr1, and B‐cr2 plants. Scale bars, 1.5 cm.

We knocked down the expression of *Gbar_A07G008330* in HaiR using virus‐induced gene silencing (VIGS). To avoid interference with other R2R3‐MYB TF, we used a tobacco rattle virus (TRV)‐based VIGS construct, which specifically targeted the third exon of *Gbar_A07G008330* (outside the conserved R2R3‐MYB domain). Two independent VIGS‐silenced cotton plants (B‐Ri1 and B‐Ri2) displayed an obviously faded or absent beauty mark phenotypes as compared with TRV‐00 control plants (Figure S4a–c). Expression analysis showed no significant alteration in the five orthologous genes between the RNAi line and wild‐type plants. But B‐Ri1 and B‐Ri2 plants displayed a significant reduction in endogenous RNA levels of other three orthologous genes, including *Gbar_A07G008300*, *Gbar_A13G011420*, *Gbar_A11G025580* compared with the control line (Figure S5). Therefore, we further generated two CRISPR‐Cas9 constructs with different single‐guide RNAs (sgRNA) and transformed them into *G. barbadense* line Xinhai25, which also exhibits the beauty mark phenotype. Briefly, the two independent homozygous lines (B‐cr1 and B‐cr2) carried null alleles resulting from a 1‐bp insertion and a 3‐bp deletion, respectively (Figure 3a), and neither line had a beauty mark phenotype (Figure 3b and Figure S6a–c). Thus, *Gbar_A07G008330* is causal gene responsible for the petal spot phenotype.

### GbBM regulates cotton flower flavonoid level

**Figure 4:**
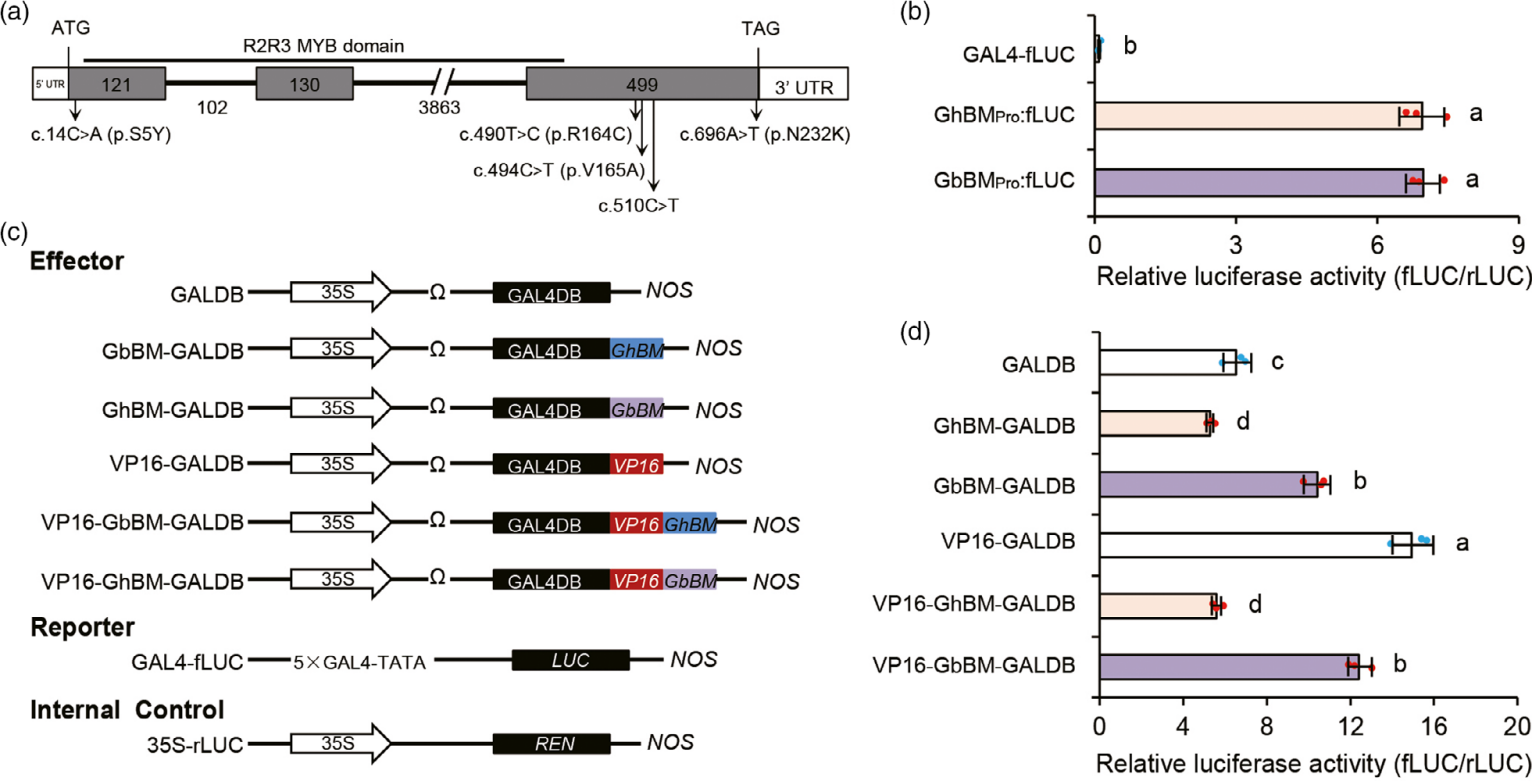
Natural variations at the coding sequence of *GhBM*. (a) Mapping of allelic variation to the *G. hirsutum GhBM* protein sequence. (b) The relative firefly/*Renilla* luciferase values of *GhBM* and *GbBM* promoter fragments in *Arabidopsis* protoplasts. The empty vector control is included as GAL4‐fLUC. Bars represent mean ± s.d. (*n* = 3 biologically independent replicates). (c) Constructs used for *GhBM* and *GbBM* transcriptional activity assays as shown in (d); VP16 was used as a positive control. TATA, TATA box for DNA binding. fLUC, firefly luciferase. REN, *Renilla* luciferase. NOS, nopaline synthase terminator. (d) Transient transcriptional activity analysis in *Arabidopsis* protoplasts illustrating the transcriptional activity of *GhBM* and *GbBM*. The relative luciferase activities were calculated by normalizing the LUC values against *Renilla*. The data in c and e were analyzed by ANOVA one‐way comparison followed by LSD test. Different letters above the bars indicate a significant difference at *P* < 0.05.

To understand the DNA sequence variation in BM, we explored DNA sequence variation for this *BM* gene, and ultimately detecting four synonymous polymorphisms (c. 14C˃A, c. 490T˃C, c. 494C˃T, and c. 696A˃T), and one missense polymorphism (c. 510C˃T) in its coding sequence (CDS) (Figure 4a), as well as five single nucleotide polymorphisms (SNPs) and three InDels in its promoter region. Using these variants, we classified *BM* gene allelic variations into two haplotypes: one with beauty mark varieties (haplotype *GbBM*) and one without beauty mark varieties (haplotype *GhBM*) (Table S4). *BM*_promoter_‐LUC assays showed no significant differences in luciferase (LUC) activity between the *GbBM*_promoter_‐LUC and *GhBM*_promoter_‐LUC constructs (Figure 4b, Figure S7, and Table S5). By contrast, LUC activity was obviously increased by transient expression of the *GbBM* CDS reporter construct, whereas *GhBM* repressed LUC activity (Figure 4c,d). These findings collectively suggest that the SNP1‐5 variations in the CDS can account for the appearance of the beauty mark.

**Figure 5:**
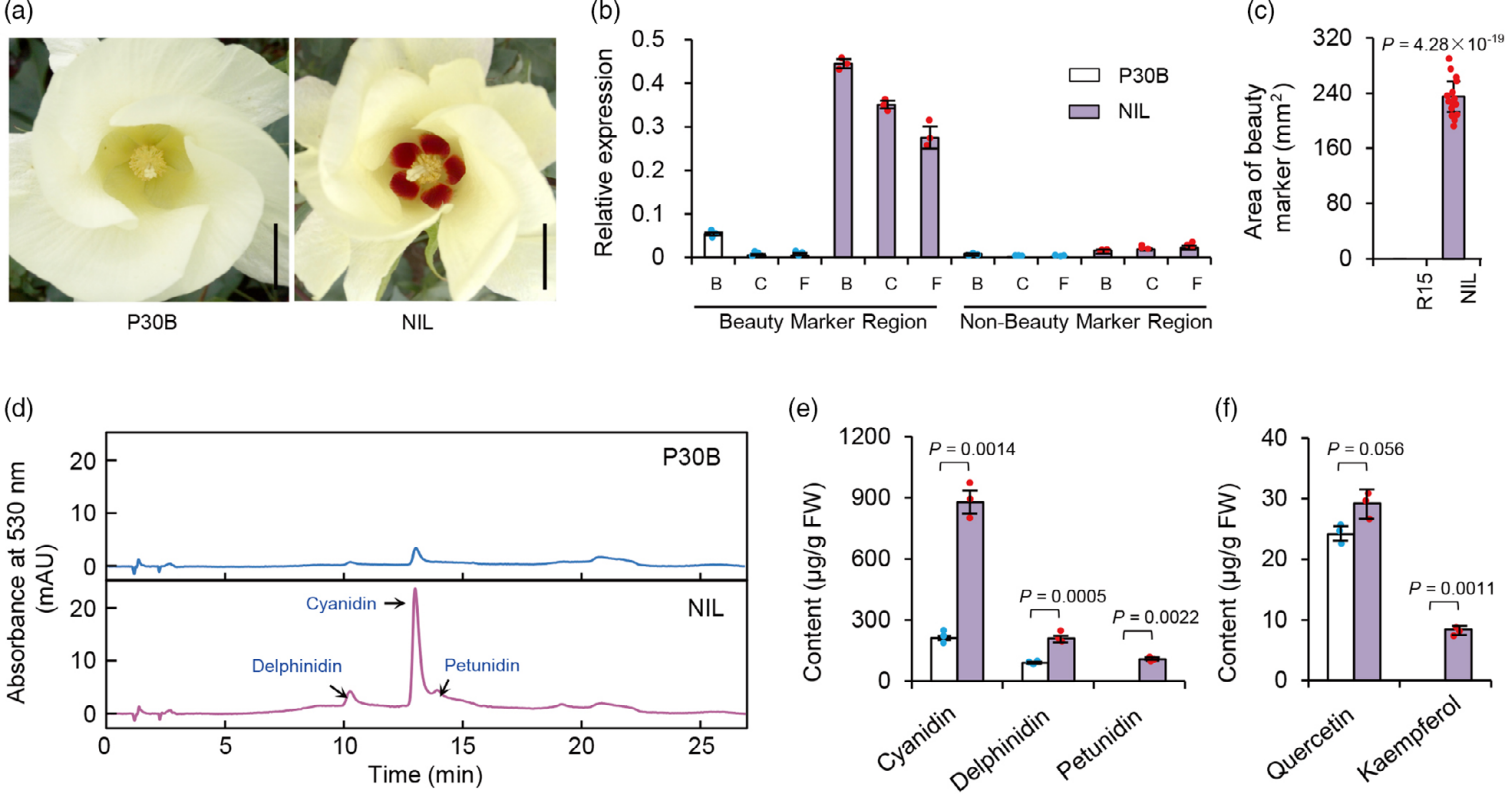
Anthocyanin and flavones content in P30B and NIL plants. (a) Gross morphologies of P30B and NIL plants at flowering stage. Scale bar, 1.5 cm. (b) Relative expression of *GbBM* gene in flower beauty mark and nonbeauty mark regions of P30B and NIL. B, bud stage. C, candle stage. F, flowering stage. The transcript levels are expressed relative to that of cotton *Actin1* in each sample and Bars represent means SD of three biological replicates. (c) Statistical analysis of area of beauty mark of P30B and NIL lines. Data are means SD (*n* = 10 flower petals). *P* values are based on two‐tailed, two‐sample *t* tests. (d). HPLC chromatograms and characteristic absorbance spectra of the extracted anthocyanin from the beauty mark region of P30B and NIL. Peaks for cyanidin, delphinidin, and petunidin were marked. mAU, milli‐absorbance unit. (e) Contents of cyanidin, delphinidin, and petunidin in beauty mark regions of the P30B and NIL. FW, fresh weight. Bars represents means SD of three biological replicates. (f) Contents of quercetin and kaempferol in nonbeauty mark regions of the P30B and NIL. FW, fresh weight. Bars represents means SD of three biological replicates. *P* values are based on two‐tailed, two‐sample *t* tests

We next generated a near‐isogenic line (NIL) carrying the *GbBM* allele from the HaiR allele in the background of the *G. hirsutum* line P30B. Compared with P30B, the NIL displayed a petal spot pattern that resembled the beauty mark phenotype of HaiR (in both color and size) (Figure 5a–c). Further, the NIL had significantly higher *GbBM* levels in its flower beauty mark region compared with P30B (Figure 5b). We then quantified the flavonoid amount in flower petal samples from both the NIL and P30B using HPLC. Compared with P30B, the NIL beauty mark region had higher levels of anthocyanins including cyanidin, delphinidin, and petunidin (Figure 5d). Specifically, cyanidin and delphinidin were 4.3‐ and 2.4‐fold higher, respectively, in the NIL compared with P30B (Figure 5e). Petunidin was detected in the beauty mark region of the NIL but not in P30B (Figure 5e). We also noted that the content of the flavonol kaempferol was dramatically increased in other petal regions (*i.e*., nonspot) of the NIL (Figure 5f).

**Figure 6:**
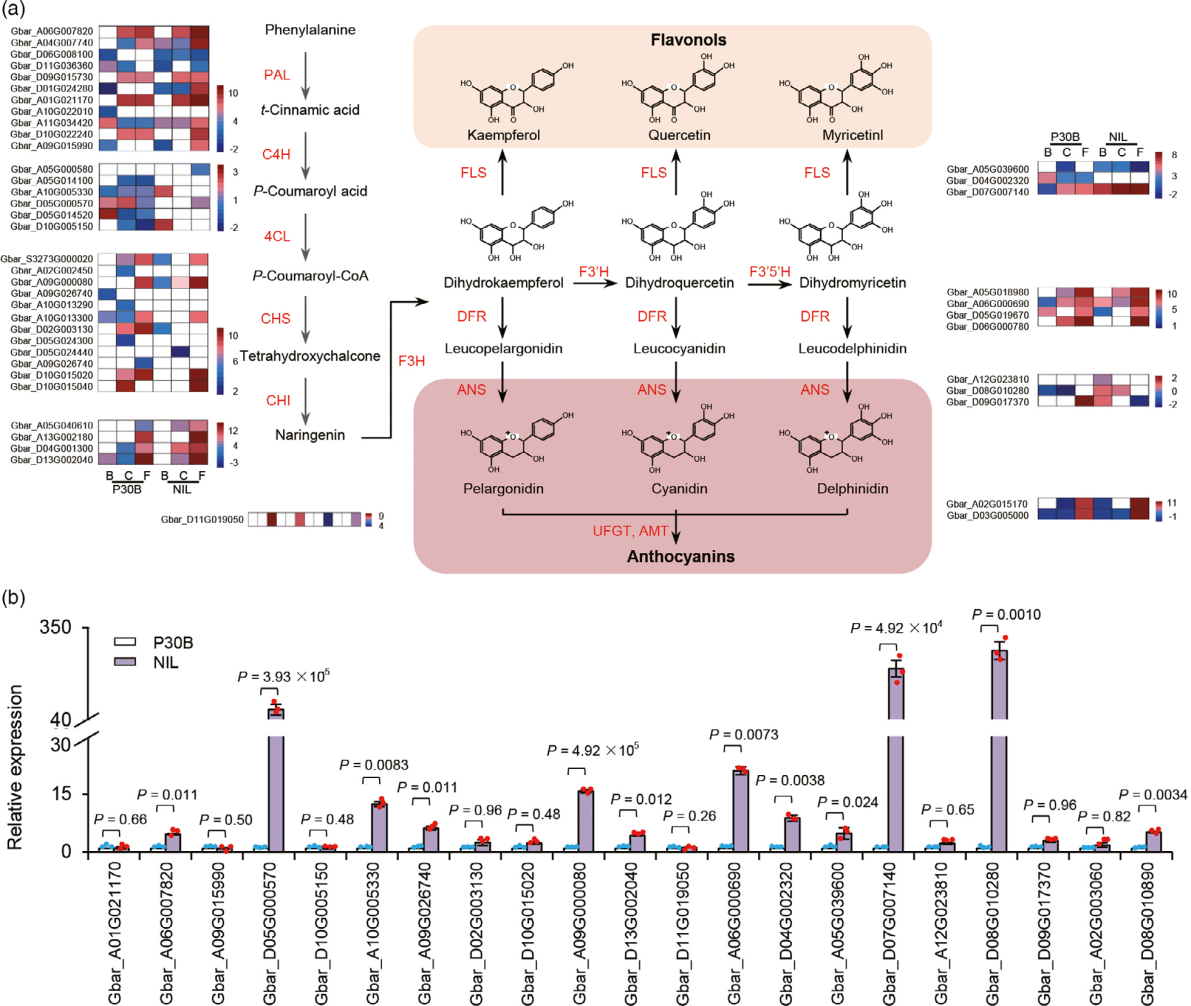
Genes of anthocyanin and flavone pathway enzymes and their expressions. (a) Genes of the enzymes catalyzing the defined steps in anthocyanin and flavones and their homologs are indicated. The transcripts levels are shown by heatmap, estimated using Cuffdiff by computing the fragment per kilobase of transcript per million reads sequence (FPKM) value for each transcript. PAL, phenylalanine ammonia lyase; C4H, cinnamate‐4‐hydroxylase; 4CL, 4‐coumarate‐coA ligase; CHS, chalcone synthase; CHI, chalcone‐flavonone isomerase; F3H, flavanone 3ß‐hydroxylase; F3’H, flavonoid 3’‐momooxygenase; F3’5’H, flavonoid 3’‐momooxygenase; FLS, flavonol synthase/flavanone 3‐hydroxylase; DFR, dihydroflavomol‐4‐reductase; ANS, anthocyanidin synthase; UFGT, UDP‐glycose flavonoid glycosyltransferase. Distributions of genes in the *G. barbadense* genome are indicated by their accession numbers. (b) Analysis of gene expression in anthocyanin and flavones pathway by qRT‐PCR. Bars represent means SD of three technical replicates. P values are based on two‐tailed, two‐sample *t* tests

RNA‐seq profiling of the beauty mark regions of NIL and P30B flowers identified 2295 differentially expressed genes (DEGs, with ≥2‐fold change) common among the DEGs for the bud (8328), candle (8084), and flower stage samples (10 541) (Table S6). Functional enrichment analysis of these common DEGs identified ‘secondary metabolites’ as top‐ranking enriched pathway (Table S6). Many DEGs were annotated as involved in flavonol biosynthesis, including 11 genes encoding PAL (Phenylalanine ammonia lyase), 6 genes encoding 4CL (4‐coumarate‐CoA ligase), 13 genes encoding CHS (chalcone synthase), 4 genes encoding CHI (chalcone isomerase), 1 gene encoding F3H (flavanone 3‐hydroxylase), 4 genes encoding FLS (flavanol synthase), 4 genes encoding DFR (dihydroflavonol 4‐reductase), 3 genes encoding ANS (anthocyanidin synthase), and 2 genes encoding UFGT (UDP‐glycose flavonoid glycosyltransferase) (Figure 6 and Table S6).

**Figure 7:**
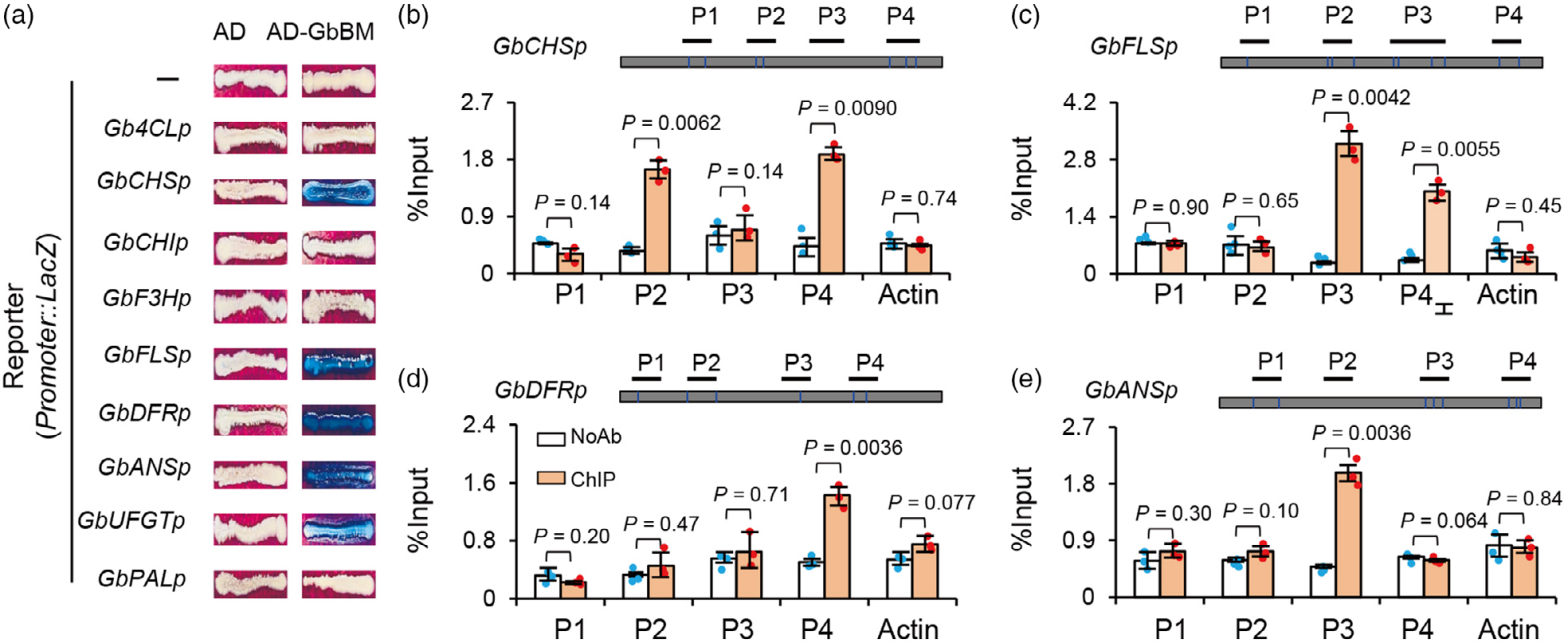
GbBM directly activates the expression of anthocyanin and flavone biosynthesis genes in *G. barbadense*. (a) AD‐GbBM activates the expression of the *lacZ* reporter genes driven by the promoters of *Gb4CL*, *GbCHS*, *GbCHI*, *GbF3H*, *GbFLS*, *GbDFR*, *GbANS*, *GbUFGT*, and *GbPAL* in yeast. Representative data are shown from one of three biological replicates, which yielded similar results. (b–e) ChIP assays indicating the association of GbBM with several regions in the promoters of *GbCHS* (b), *GbFLS* (c), *GbDFR* (d), and *GbANS* (e). The regions tested by ChIP assays are shown in the schematic representation. The putative MYB‐core elements are indicated by black lines, respectively. The promoter of *GbActin1* was used for normalization. Bars represent means SD of three biological replicates. *P* values are based on two‐tailed, two‐sample *t* tests.

A yeast one‐hybrid analysis showed that a GAL4 transcriptional activation domain‐GbBM (AD‐GbBM) could directly bind and activate the *LacZ* reporter gene driven by the promoters of *GbCHS* (*Gbar_A09G000080*), *GbDFR* (*Gbar_A06G000690*), *GbANS* (*Gbar_D08G010280*), *GbUFGT* (*Gbar_D08G010890*), and *GbFLS* (*Gbar_D07G007140*) (Figure 7a), respectively. And chromatin immunoprecipitation with quantitative PCR (ChIP‐PCR) assays revealed significant enrichment for *GbCHS*, *GbDFR*, *GbANS*, and *GbFLS* promoter fragments containing the MYB core motifs in the GbBM‐FLAG chromatin samples (Figure 7b–e). These results indicate that GbBM can directly bind to the promoters of *GbCHS*, *GbDFR*, *GbANS*, and *GbFLS*, each of which encode flavonol biosynthesis enzymes. Thus, the R2R3‐MYB TF protein GbBM functions is specifically expressed in flower petal beauty mark region and functions as a positive transcriptional regulator of flavonoid biosynthesis genes.

### *GbBM* introgression significantly increases honeybee visiting rate

**Figure 8:**
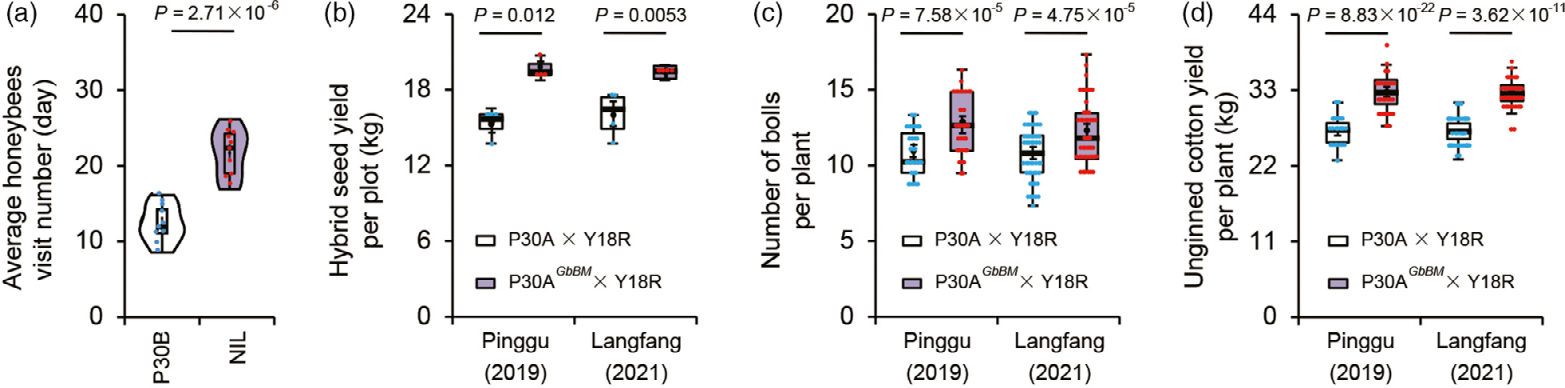
Reshaping of flower beauty mark contributes to honeybee visiting frequency reinforcement and increases hybrid seed yield in *G. hirsutum*. (a) Average honeybees visit number for a flower per day was evaluated using honeybees in an experimental greenhouse. Value is mean SD of ten days. (b–d) Agronomic traits of P30A and P30A*^GbBM^* after crossing with Y18R by honeybee‐mediated pollination. Hybrid seed yield per plot (b) (*n* = 4 plots), boll numbers (c) (*n* = 50 plants), and unginned cotton yield per plant (d) (*n* = 50 plants) of P30A and P30A*^GbBM^*. In a–d, *P* values are based on two‐tailed, two‐sample *t* tests

Honeybees are known to be the primary pollinators responsible for outcrossing in cotton fields (Moffet and Stith, 1972, 1978; Vaissiere and Moffet, 1984; Waller *et al*., 1985; Xing *et al*., 2005). We next explored whether the beauty mark phenotype promotes the frequency of honeybee visitation with experiments wherein honey bees were offered a choice between NIL and P30B flowers. We found that honeybees preferentially selected NIL flowers as their first choice. Briefly, the NIL flowers received approximately twice as many feeding event visitations as the P30B flowers (Figure 8a).

### GbBM confers the potential for improving hybrid seed yield

We evaluated whether the presence of the beauty mark may improve hybrid seed yields in *G. hirsutum* in the field through the marker‐assisted introgression of *GbBM* into P30A, an elite *G. hirsutum* cytoplasmic male sterile line widely used in three‐line hybrid cotton breeding in China, generating the near isogenic line P30A*^GbBM^*. We carried out large‐scale agronomic trait evaluation of both P30A*^GbBM^* and P30A after crossing these lines with the *G. hirsutum* restorer line Y18R through honeybee‐mediated pollination for two successive years (2019 in Pinggu and 2021 in Langfang). The field test data showed that the increase in hybrid seed yield per plot was 14.9% (Figure 8b), the boll number per plant was significantly increased in the P30A*^GbBM^* lines (Figure 8c), and the P30A*^GbBM^* plants had a 19.7% increase in the unginned cotton yield per plant (Figure 8d). No differences in boll weight, lint percentage, micronaire, fiber length, uniformity, fiber strength, and breaking elongation were detected between P30A*^GbBM^* and its corresponding recipient parent P30A (Figure S8 and S9). These results establish that the introduction of the *GbBM* allele to an elite *G. hirsutum* hybrid breeding line increases both cotton and hybrid seed yields, ostensibly by attracting more honeybees and therefore increasing the number of pollination events.

## Discussion

In this study, we found that *G. barbadense*, a close relative of *G*. *hirsutum*, has purple spot at the base of flower petals which significantly increases the number of honeybee visiting and improves pollination. Here, we cloned the *Beauty Mark* (*GbBM*) gene from *G. barbadense* by map‐based cloning method, and demonstrated that *GbBM* could activate anthocyanin production, and directly led to formation of a pollinator‐attracting purple spot at the base of flower petals. We confirmed that the presence of these spots significantly increases the number of honeybees visiting and improves pollination by introducing the *GbBM* allele from *G. barbadense* to *G. hirsutum*. We further demonstrated that hybrid seed production was significantly increased in the main commercial cotton species *G*. *hirsutum* using marker‐assisted introgression.

Given its apparent benefit to reproductive success, it is somewhat puzzling that the beauty mark trait was apparently lost in most *G. hirsutum* varieties. In this regard, it bears emphasis that several previously reported QTLs for fiber yield or quality have been mapped to a genomic near the *Beauty Mark* gene on chromosome A07 (Fang *et al*., 2017; Ma *et al*., 2018). Perhaps loss of *GhBM* function was linked during the selection of some valuable agronomic trait during the domestication, artificial selection, and/or on‐going breeding of *G. hirsutum*. Whatever the genetic history leading to the current lack of petal spots in most *G. hirsutum* varieties, our study has demonstrated that “retrieval” of the beauty mark trait in *G. hirsutum* flowers (based on marker‐assisted introgression of an allele from the closely related tetraploid species *G. barbadense*) enhances the extent of insect‐mediated pollination and increases hybrid seed yield. This approach can be harnessed to reduce costs and increase the efficiency of cotton hybrid seed production, which can ultimately promote the broader adoption of elite, high‐yield hybrid cotton lines.

## Materials and methods

### Plant materials

The *G. barbadense* line HaiR and *G. hirsutum* line P30A and P30B were used in this study. P30A is a *G. hirsutum* cytoplasmic male sterile line, P30B is a *G. hirsutum* maintainer of P30A, Y18R is a *G. hirsutum* restorer of P30A, and HaiR is a *G. barbadense* restorer of P30A (Shi *et al*., 2012). *GbBM* gene‐edited plants were generated in the *G. barbadense* line Hai7124 background. The *G. hirsutum* line P30B × *G. barbadense* line HaiR F_1_ was backcrossed eight times with P30B to generate the near‐isogenic line (NIL). P30A*^GbBM^* were developed by backcrossing with NIL in the P30A background.

### Cotton cultivation conditions

For genetic analysis, cotton plants were cultivated under field conditions at the experimental stations of the Biotechnology Research Institute (BRI) at the Chinese Academy of Agricultural Sciences (CAAS) in Pinggu, (Beijing, 39°56′N, 116°20′E) and Langfang (Hebei province, N39°52′, E116°70′E). Large‐scale field tests of P30A and P30A*^GbBM^* lines were performed during the regular cotton cultivation season in 2019 at the experimental stations of the BRI, CAAS in Beijing. Two ridges of the P30A and P30A*^GbBM^* intercrop one ridge of restorer lines Y18R. The plant spacing was 30 cm, the row spacing was 90 cm, and the plot size for yield tests was 120 m^2^. One hive for the honeybee was put in the middle of each plot during the flowering time. Four replicates were used for plot‐yield assays.

### Map‐based cloning

Map‐based cloning was performed with an interspecific F_2_ population derived from P30B and HaiR. About 5660 segregants without the beauty mark phenotype were selected at the blooming stage for genetic linkage analysis. A total of 720 pairs of insertion/deletion (InDel) primers were designed based on the genome‐wide InDel (>80 bp) between *G. hirsutum* and *G. barbadens*e genotypes. Genome‐wide sequencing data from *G. hirsutum* and *G. barbadens*e released by Nanjing Agricultural University was downloaded from NCBI's SRA database (https://www.ncbi.nlm.nih.gov/sra). The PCR product was electrophoresed on a 4% agarose gel. Primers used for fine mapping are listed in Table S2.

### Virus‐induced gene silencing assays

A 330 bp fragment of *GbBM* was amplified from cDNA using VIGS primers (Table S2). The PCR products digested with *Eco*RI and *Bam*HI were inserted into *Eco*RI‐*Bam*HI‐cut pTRV2. The pTRV1 and pTRV2 derivatives fused to the target fragment of *GbBM* vectors were transformed into *Agrobacterium tumefaciens* strain LBA4404. The transformed *Agrobacterium* cells were collected and resuspended in infiltration medium, and the cell suspensions were adjusted to an OD_600_ = 1.5. *Agrobacterium* cultures containing pTRV1 and pTRV2 or its derivative vectors were mixed at a ratio of 1:1. Leaves closest to the 1‐day bud were infiltrated with the *Agrobacterium* mixed suspension using a needle‐less syringe. Plants were left at room temperature under dim light for 12 h. Primers used for vector construction are listed in Table S2.

### RNA extraction, cDNA preparation, and gene expression analysis

For expression analysis, various tissues were collected and immediately flash frozen in liquid nitrogen, then stored at −80 °C for RNA extraction. Total RNA was extracted using a TRIzol kit according to the manufacturer’s instructions (Invitrogen, Carlsbad, CA). RNA was reverse transcribed using the ReverTra Ace qPCR RT Master Kit (Toyobo, Japan). Quantitative real‐time (qRT)‐PCR was performed with a Chromo 4 real‐time PCR detection system following the manufacturer’s instructions (Bio‐Rad, CFX96, Hercules, USA). The data were analyzed using Opticon monitor software (Bio‐Rad, Hercules, USA). Cotton *GhAct1* was used as an internal reference gene. All experiments were repeated three times independently, and the representative results were presented. Primers used for qRT‐PCR are listed in Table S2.

### RNA sequencing and data analysis

RNAs extracted from beauty mark and nonbeauty mark regions of flower petals were used for RNA sequencing. For direct comparison, two libraries (150 bp‐length paired‐end reads) were prepared in the same manner and sequenced on the Hiseq2000 platform (Biomarker, Beijing, China) following the protocol provided by the company (Illumina, San Diego, CA). After removal of adaptor sequences, duplicated sequences, ambiguous reads, and low‐quality reads, high‐quality clean reads were generated and separately mapped to the *G. hirsutum* and *G. barbadens*e genomes using Bowtie software (Langmead and Salzberg, 2012). Differentially expressed genes were analyzed by Cufflinks software for FPKM (fragments per kilo bases per million reads) calculation (Trapnell *et al*., 2010). Genes with more than 2‐fold change and *P* values ≤ 0.05 were regarded as DEGs. Enrichment analysis was performed using in‐house Perl scripts with known gene function annotations downloaded from PlantGSEA provided in Cytoscape (Yi *et al*., 2013).

### CRISPR/Cas9 construct design and cotton transformation

Two *GbBM* target sites (sgRNA1 and sgRNA2) of 20 nucleotides (nt) were manually selected. To confirm specificity, the sgRNA sequences were aligned to the whole cotton genome, and no potential sites with mismatches over six bases were found. The CRISPR/Cas9 constructs were generated following a published description (Wang et al., 2017b). The sgRNA sequences were incorporated into two 60 nt oligonucleotides (sgRNA1‐Forward: GAAGCTGAGTTTATATACAGCTAGAGTCGAAGTAGTGATTGCCAGCTTGTGATAGTGCCA, and Reverse: GACTAGCCTTATTTTAACTTGCTATTTCTAGCTCTAAAACTGGCACTATCACAAGCTGG; sgRNA2‐Forward: GAAGCTGAGTTTATATACAGCTAGAGTCGAAGTAGTGATTGGAAGATCTAGCAAAGATAG, and Reverse, GACTAGCCTTATTTTAACTTGCTATTTCTAGCTCTAAAACCTATCTTTGCTAGATCTTCC). The two primers were annealed and extended to make 100‐bp double‐stranded DNA fragments, and then cloned into NcoI‐linearized pMR093 vector with the In‐Fusion cloning kit (Clontech). The plasmid with the correct insertion was introduced into *A. tumefaciens* strain GV3101 for *G. barbadens*e transformation. The CRISPR/Cas9 vectors were transformed into *G. barbadens*e with the cotton shoot tip‐*Agrobacterium* transformation method following a previous study (Chen *et al*., 2014).

### Transient luciferase assay

The transient transcriptional activity analysis assay was performed as described previously (Wang et al., 2017a). The coding sequences of *GbBM* and *GhBM* were cloned into the effector GAL4‐DB vector. Two types of reporters were used, one containing the LUC gene fused with the 5× GAL4‐binding site and the other a plasmid expressing the REN gene as the internal control. The effector plasmids (*GAL4‐DB‐GbBM, GAL4‐DB‐GhBM*, *GAL4‐DBVP16‐GbBM*, *GAL4‐DBVP16‐GhBM*, or positive control GAL4‐DBVP16) were co‐transformed with two reporters and the activities of LUC and REN were separately determined 18 h post‐transformation using Dual‐Luciferase^®^ Reporter Assay System (Promega, E1910). For the transient transcriptional activity assays on specific promoters, the *GhBM* or *GbBM* promoter sequences were amplified from HaiR and P30A genome DNA, respectively, and cloned into the pGreenII 0800‐LUC vector as a reporter. The REN gene under the control of the cauliflower *35S* promoter in the pGreenII 0800‐LUC vector was used as the internal control. The coding sequences of *GhBM* or *GbBM* were cloned into the p2GW7 vector under the control of the 35S promoter and were used as effectors. The reporters and effectors were transformed to *Agrobacterium* strain GV3101 and then co‐infiltrated into *N. benthamiana* leaves, and the LUC activities were analyzed after 48‐h infiltration using NightSHADE LB 985 (Berthold Technologies, USA).

### Yeast one‐hybrid assay

To generate AD‐GbBM, the full‐length *GbBM* was amplified using KOD polymerase (Toyobo, Japan), and subcloned into the *Eco*RI and *Xho*I sites of the binary vector pJG4‐5 vector (Clontech). To generate *GbPALp::LacZ*, *Gb4CLp::LacZ*, *GbCHSp::LacZ*, *GbCHIp::LacZ*, *GbF3Hp::LacZ*, *GbFLSp::LacZ*, *GbDFRp::LacZ*, *GbANSp::LacZ*, and *GbUFGTp::LacZ*, reporter constructs, the promoter fragments were amplified by PCR and then separately cloned into the pLacZi2μ vector. The primer sequences are listed in Table S2. Transformants were grown on proper drop‐out plates containing X‐gal (5‐bromo‐4‐chloro‐3‐indolyl‐β‐D‐ galactopyranoside) for blue color development. Representative data was shown from one of the three biological replicates, which yielded similar results.

### Chromatin immunoprecipitation assays

The wild‐type *G. barbadens*e line HaiR and its transgenic calli harboring the *35S:GbBM‐MYC* construct were used for ChIP assays according to the method described previously (Kaufmann *et al*., 2010). Approximately 2 g of each calli was harvested and cross‐linked in 1% formaldehyde for 15 min, followed by 5‐min neutralization with 0.125 m of glycine. After washing five times with distilled water, the samples were ground to powder in liquid nitrogen and the chromatin complexes were isolated and sonicated and then the chromatin complex was immunoprecipitated by anti‐Myc antibody (MBL, catalog number M185‐3). The precipitated DNA was recovered and analyzed with real‐time qPCR using the primers included in Table S2. The promoter of *Ubiquitin* was employed as a negative control. Three independent biological repeats were performed.

### Subcellular localization

Full‐length *GbBM* coding sequence was amplified using primers listed in Table S2, and inserted into *CaMV 35S*::eGFP vector. The binary vector *CaMV 35S::GbBM*‐*eGFP* was subsequently transformed into *Arabidopsis* protoplasts using the polyethylene glycol method. After overnight incubation in the darkness, the protoplasts expressing eGFP were imaged by a confocal laser scanning microscope (LSM510, Zeiss, Germany). Composite figures were prepared using Zeiss LSM Image Browser software.

### Anthocyanin analysis

Anthocyanin content was determined as described previously (Pang *et al*., 2009). Dissected petals (10 mg) from fully opened flowers were ground into a powder in liquid nitrogen and extracted with 0.1% (v/v) HCl/methanol solution. Samples were sonicated for 15 min and hydrolyzed at 100 °C for 10 min to obtain aglycones. Following centrifugation at 15,000 × g, the supernatant was diluted 1:1,000 with methanol containing 1% HCL. LC‐MS analysis was carried out using an Agilent 1260 HPLC System linked to a QTRAP 5500 mass spectrometer. Peak areas of each compound for all samples were normalized to the internal standard, and quantified by external calibration to standards, including cyanidin (Sigma‐Aldrich, Saint Louis, USA), delphinidin (Sigma‐Aldrich, Saint Louis, USA), pelargonidin (Sigma‐Aldrich, Saint Louis, USA), paeonidin (Sigma‐Aldrich, Saint Louis, USA), malvidin (Sigma‐Aldrich, Saint Louis, USA), and petunidin (Sigma‐Aldrich, Saint Louis, USA).

### Flavonoid analysis

Extraction and determination of the total flavonoid content from fully opened flowers were performed according to the method described previously (Meng *et al*., 2019) with four independent biological repeats. The samples were filtered with a 0.22‐mm syringe filter, and analyzed with an ultraperformance liquid chromatography‐tandem mass spectrometry system consisting of an Agilent 1290 Infinity LC pump and a 6495 triple‐quadrupole mass spectrometer. The content of each compound was calculated using the corresponding standard as an external standard. Flavonoid standards used were kaempferol (Sigma‐Aldrich, Saint Louis, USA), quercetin (Sigma‐Aldrich, Saint Louis, USA), myricetin, and naringenin (Sigma‐Aldrich, Saint Louis, USA).

### Pollinator behavioral experiments

Pollinator preference experiment was carried out as described previously (Dell'Olivo and Kuhlemeier, 2013), with minor modifications. Briefly, honeybees were used as pollinators and obtained from the field research station of the BRI, CAAS (Beijing). Hives were made of a plastic container protected by a cardboard box, with a sugar solution tank at the bottom. They were used for experiments from 1 to 9 days and setup was maintained in the greenhouse. Equal numbers of P30B and NIL cotton plants were used to test pollinator preference at the same stage. Observations of honeybees visit were recorded in the morning between 9:00 and 11:00, and in the afternoon between 14:00 to 16:00. On each day, visits were recorded for 15 min periods spaced evenly during measuring time. Hives were kept in the greenhouse during whole experiment, to let the bees adjust to the experimental area. The position of the plants was changed each day to minimize the impact of the first flower seen on entry. The honeybees flew freely during that time and their behaviors (visiting flower,) were recorded. We recorded the number of honeybees visit (the insect clearly touching the anthers with the head) separately for each line. Data for number of honeybees that do not pollinate the flower (merely fly over the flowers) was neglected. Visit frequency of honeybees to flowers was calculated by dividing the total number of visits recorded in a 15 min observation period by the number of flowers being observed for this time.

### Phylogenetic analysis

Protein sequences showing similarity to GbBM were retrieved using the public BLAST on the National Center for Biotechnology Information Website (http://www.ncbi.nlm.nih.gov/). Multiple alignments of the homologs was performed by Clustal X version 2.0 with the default parameters and manually adjusted. The neighbor‐joining method in MEGA 4.1 software was used to test the confidence of topology.

### Accession codes

Genes and their associated accession codes from CottonFGD are as follow: *GbBM* (*Gbar_A07G008330*), *ORF1* (*Gbar_A07G008240*), *ORF2* (*Gbar_A07G008250*), *ORF3* (*Gbar_A07G008260*), *ORF4* (*Gbar_A07G008270*), *ORF5* (*Gbar_A07G008280*), *ORF6* (*Gbar_A07G008290*), *ORF7* (*Gbar_A07G008300*), *Gb4CL* (*Gbar_D05G000570*), *GbCHS* (*Gbar_A09G000080*), *GbCHI* (*Gbar_D13G002040*), *GbF3H* (*Gbar_D11G019050*), *GbFLS* (*Gbar_D07G007140*), *GbDFR* (*Gbar_A06G000690*), *GbANS* (*Gbar_D08G010280*), and *GbUFGT* (*Gbar_D08G010890*).

## Conflict of interest

The authors declare no competing interests.

## Author contributions

C.L. and R.Z. conceived the project and designed the experiments. A.M., Y.W., Z.M., Ya.W., H.H., Q.Z., Y.L., P.W., X.L., L.Y., W.M., S.G., and C.L. performed the experiments. A.M., Yuan.W., Y.Y., R.Z., C.C., and C.L. analyzed the data. C.L., A.M., R.Z., and C.C. wrote the paper.

## Supporting information


**Figure S1** Flower phenotypes of P30B, F1 (P30B × HaiR), and HaiR at flowering time.
**Figure S2** Agarose gel electrophoresis results for mapping of the F2 population to identify *GbBM* position.
**Figure S3** Expression Analysis of candidate genes between markers CMB5 and CMB7.
**Figure S4** VIGS‐induced mutations of the *GbBM* locus in T_0_ *G. barbadense*.
**Figure S5** Expression analysis of eight orthologous members of *GbBM* among TRV‐00, B‐Ri1, and B‐Ri2 lines.
**Figure S6** Beauty mark phenotypes of the T1 *GbBM* CRISPR/Cas9 lines in wild type, B‐cr1, and B‐cr2 plants.
**Figure S7** Constructs used for *GhBM* and *GbBM* promoter analysis.
**Figure S8** Agronomic traits of P30A and P30A*^GbBM^* after crossing with Y18R by honeybee‐mediated pollination.
**Figure S9** Fiber quality traits of P30A and P30A*^GbBM^* after crossing with Y18R by honeybee‐mediated pollination.
**Table S1** Genetic analysis using F_2_ population derived with *G. hirsutum* line P30A and *G. barbadense* line HaiR.
**Table S2** Primers used in this study.
**Table S3** Candidate ORFs between the mapping markers CMB5 and CMB7.
**Table S4** Haplotypes of *Beauty mark* between *G. hirsutum* line P30B and *G. barbadense* line HaiR.

**Table S5** Comparison analysis of cis‐acting regulatory elements between *GbBM* and *GhBM* promoter.

**Table S6** RNA‐Seq data.
