# Supplementary material for: Increasing floral visitation and hybrid seed production mediated by beauty mark in Gossypium hirsutum
Source: Plant Biotechnol J. 2022 Mar 22;20(7):1274–84. doi: 10.1111/pbi.13805 (PMC9241374; doi:10.1111/pbi.13805)
Supplement: Supplementary file 1 — Figure S1 Flower phenotypes of P30B, F1 (P30B × HaiR), and HaiR at flowering time. Figure S2 Agarose gel electrophoresis results for mapping of the F2 population to identify GbBM position. Figure S3 Expression Analysis of candidate genes between markers CMB5 and CMB7. Figure S4 VIGS‐induced mutations of the GbBM locus in T0 G. barbadense. Figure S5 Expression analysis of eight orthologous members of GbBM among TRV‐00, B‐Ri1, and B‐Ri2 lines. Figure S6 Beauty mark phenotypes of the T1 GbBM CRISPR/Cas9 lines in wild type, B‐cr1, and B‐cr2 plants. Figure S7 Constructs used for GhBM and GbBM promoter analysis. Figure S8 Agronomic traits of P30A and P30A GbBM after crossing with Y18R by honeybee‐mediated pollination. Figure S9 Fiber quality traits of P30A and P30A GbBM after crossing with Y18R by honeybee‐mediated pollination. Table S1 Genetic analysis using F2 population derived with G. hirsutum line P30A and G. barbadense line HaiR. Table S2 Primers used in this study. Table S3 Candidate ORFs between the mapping markers CMB5 and CMB7. Table S4 Haplotypes of Beauty mark between G. hirsutum line P30B and G. barbadense line HaiR. [file PBI-20-1274-s001.docx]

**Supporting Information**

**
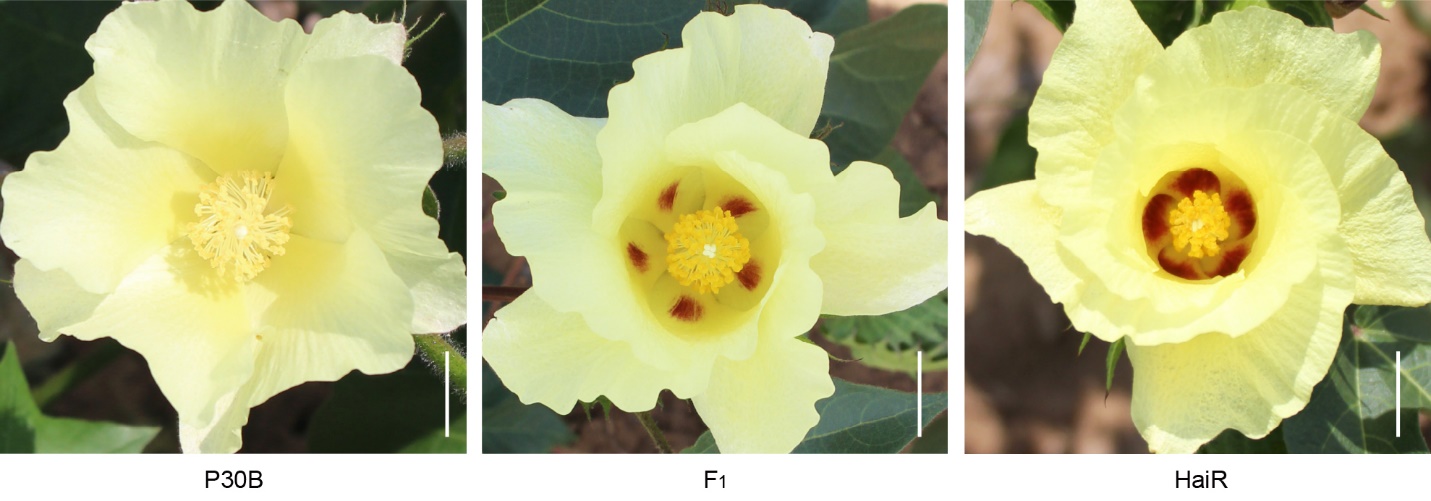
**

**Figure S1 Flower phenotypes of P30B, F_1_ (P30B × HaiR), and HaiR at flowering time.** Scale bars, 1 cm.

**
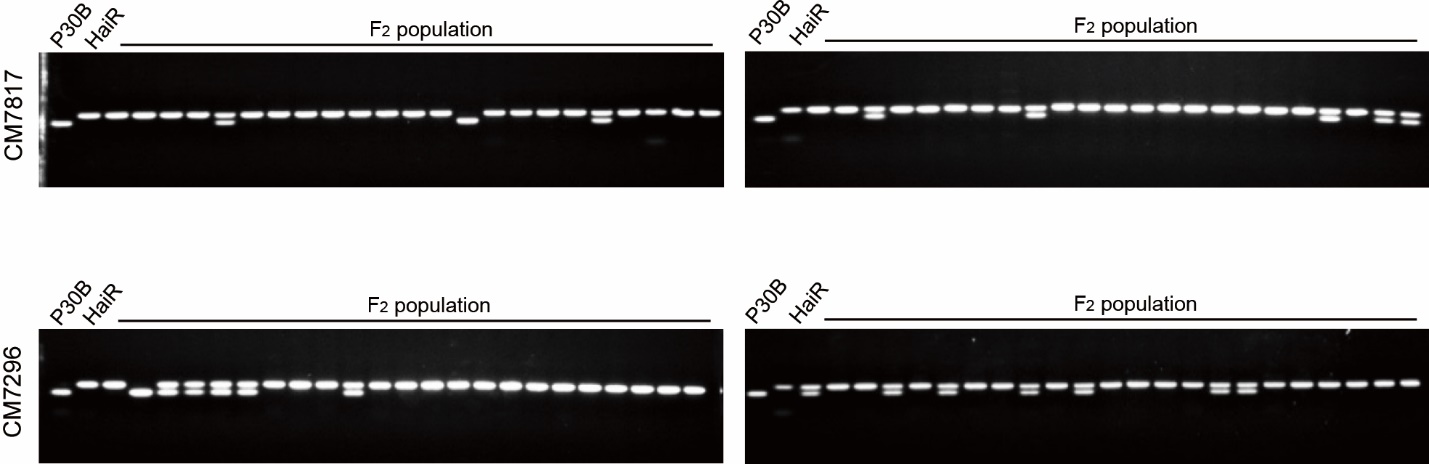
**

**Figure S2 Agarose gel electrophoresis results for mapping of the F_2_ population to identify *GbBM* position.**

**
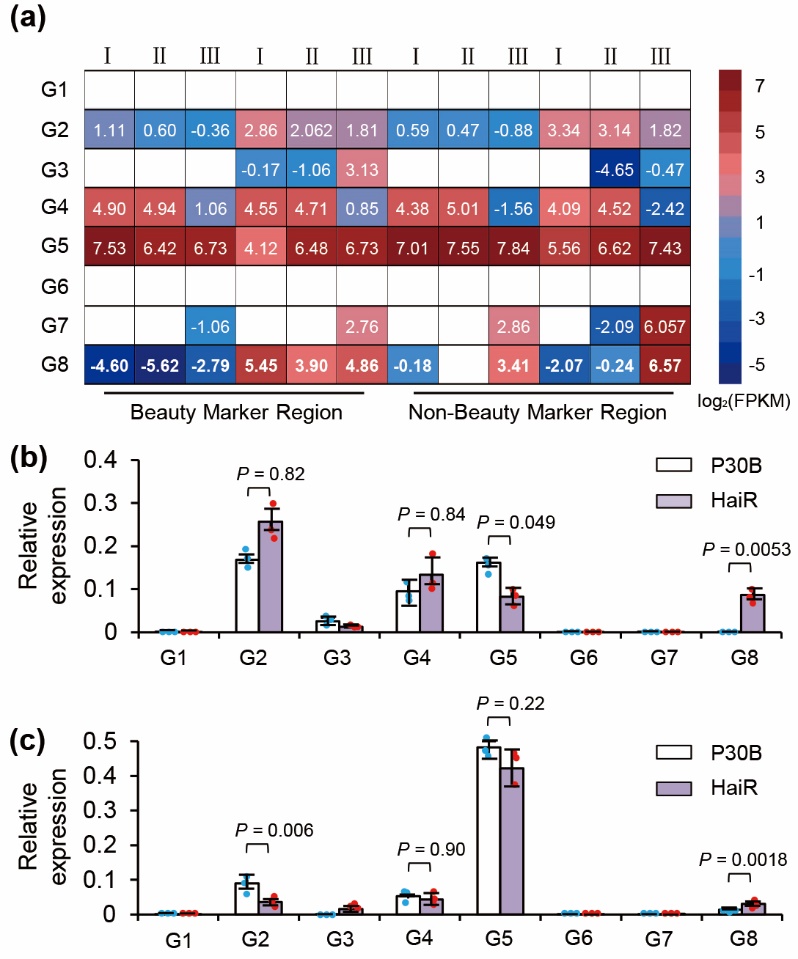
**

**Figure S3 Expression analysis of candidate genes between markers CMB5 and CMB7. (a)** A heatmap of the RNA-seq expression data for three candidate R2R3 MYB transcription factor genes. The color key (blue to red) shows gene expression (fragments per kilobase per million mapped reads (FPKM)). For each gene, the lowest FPKM value was set as 1.00). I, bud stage. II, candle stage. III, flowering stage. nd, not detected. **(b)** qRT-PCR analysis of the transcript levels of candidate genes (G1-8) in the beauty mark region of HaiR and P30B during the flowering stage. **(c)** qRT-PCR analysis of the transcript levels of G1-8 in the non-beauty mark region of HaiR and P30B during the flowering stage. Bars represent means SD of three technical replicates. *P* values are based on two-tailed, two-sample *t* tests.

**
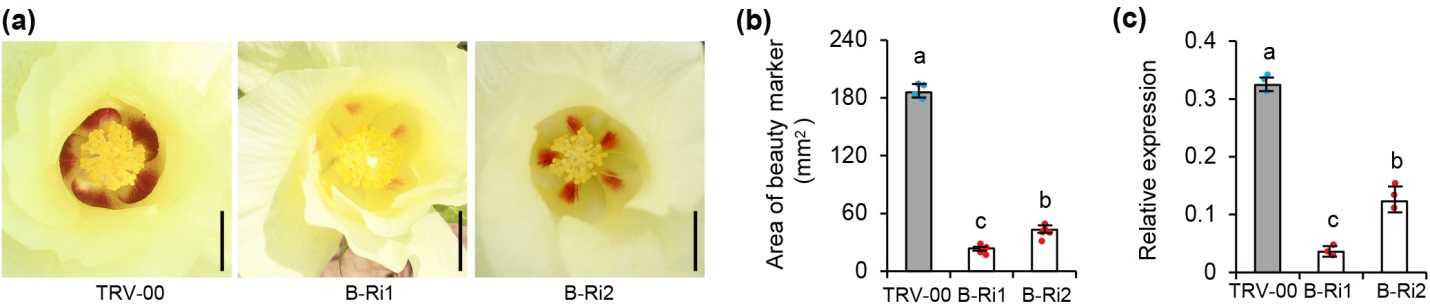
Figure S4 VIGS-induced mutations of the *GbBM* locus in T_0_ *G. barbadense*.** **(a)** VIGS in *Gossypium barbadense* flowers for comparison of flower beauty mark phenotypes in the TRV-00 (control vector) plants and B-Ri1 and B-Ri2 (*GbBM*-silenced) plants. Scale bars, 1.5 cm. **(b)** Quantitative analysis of the beauty mark area of TRV-00, B-Ri1, and B-Ri2 flowers*.* Data are means ± SD (n = 10 flower petals). **(c)** *GbBM* expression in TRV-00, B-Ri1, and B-Ri2 flowers (all *G. barbadense*). Data are means ± SD (n = 3 technical replicates). The data in **(b)** and **(c)** were analyzed by one-way ANOVA followed by an LSD test. Different letters above the bars indicate a significant difference at *P* < 0.05.


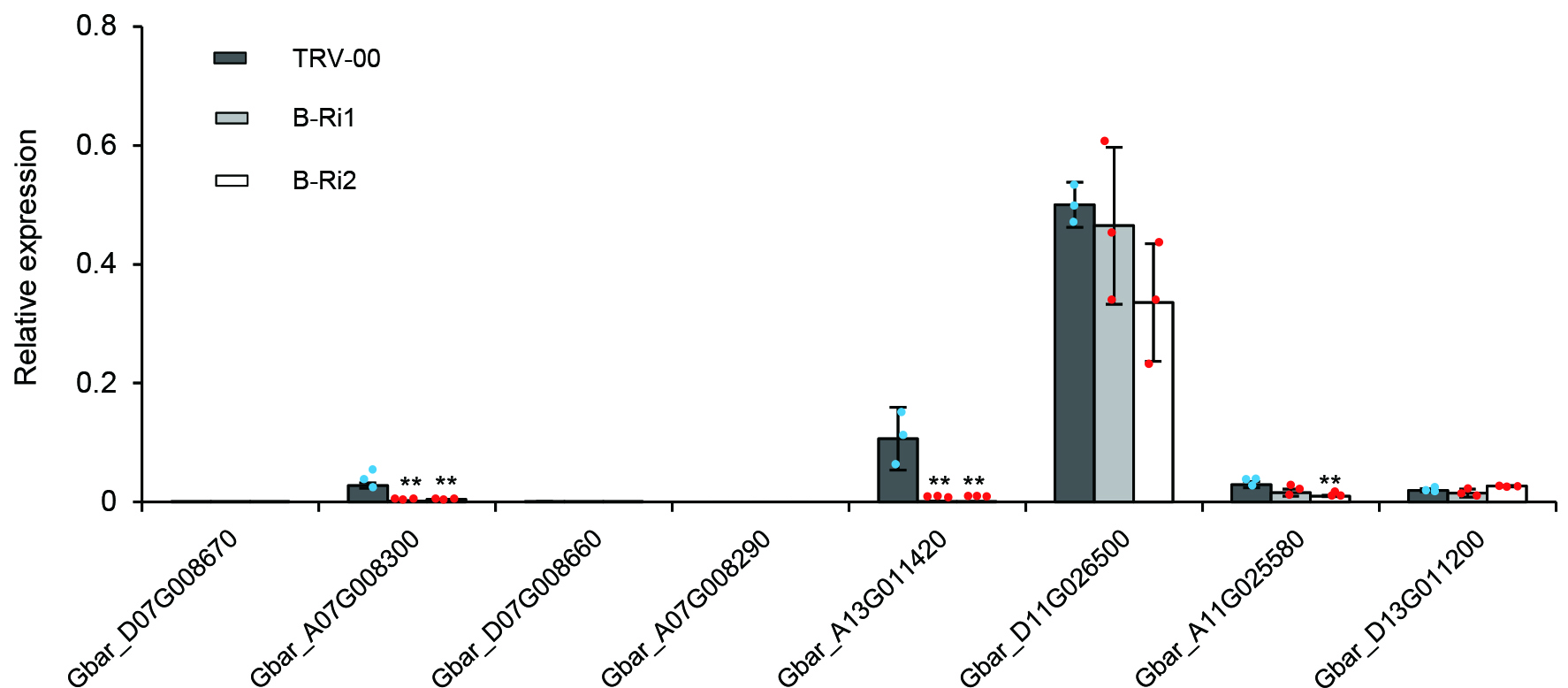


**Figure S5 Expression analysis of eight orthologous members of *GbBM* among TRV-00, B-Ri1, and B-Ri2 lines.** *Gbar_D07G008670*, *Gbar_D07G008660*, and *Gbar_A07G008290* were not detected in flower petal spotted region. Data are means ± SD (n = 3 technical replicates). Bars represent means SD of three technical replicates. *P* values are based on two-tailed, two-sample *t* tests.

**
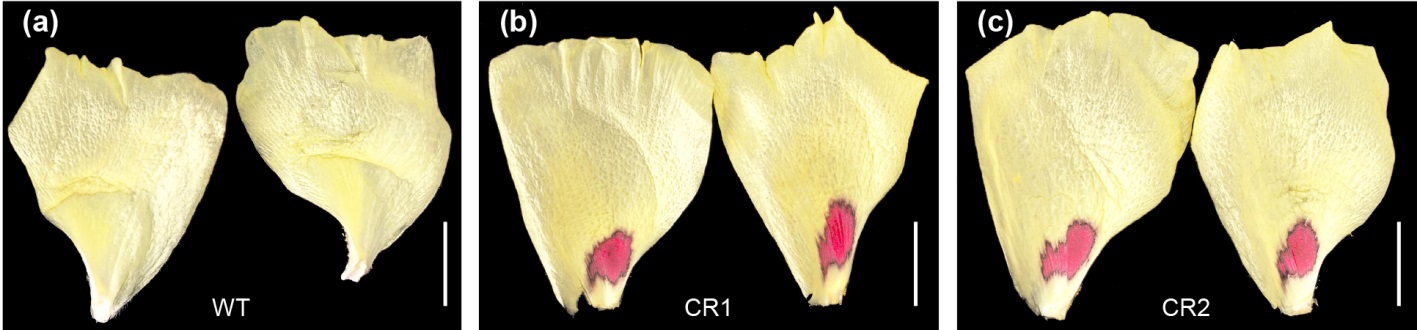
**

**Figure S6 Beauty mark phenotypes of the T_1_ *GbBM* CRISPR/Cas9 lines in wild type, B-cr1, and B-cr2 plants.** Scale bars, 1.5 cm.

**
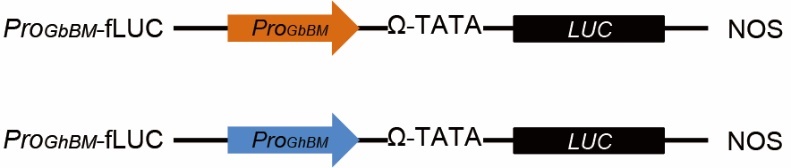
**

**Figure S7**  **Constructs used for *GhBM* and *GbBM* promoter analysis.** TATA, TATA box for DNA binding. LUC, firefly luciferase. NOS, nopaline synthase terminator.

**
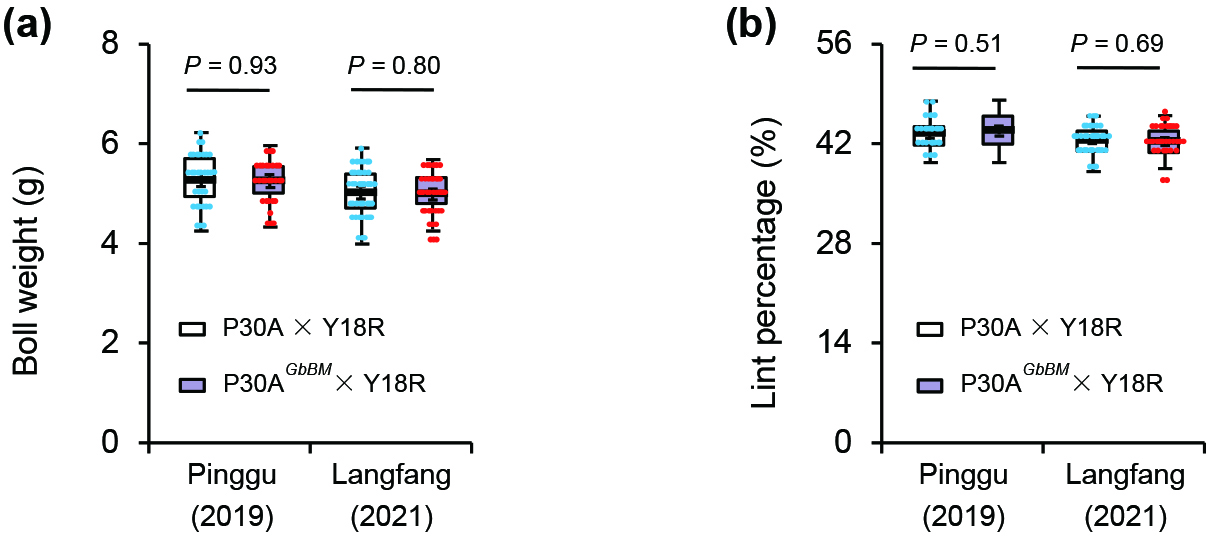
**

**Figure S8 Agronomic traits of P30A and P30A*^GbBM^* after crossing with Y18R by honeybee mediated pollination.** Boll weight **(a)** (*n* = 50 plants) and lint percentage **(b)** (*n* = 10 plants) of P30A and P30A*^GbBM^*. *P* values are based on two-tailed, two-sample *t* tests.

**
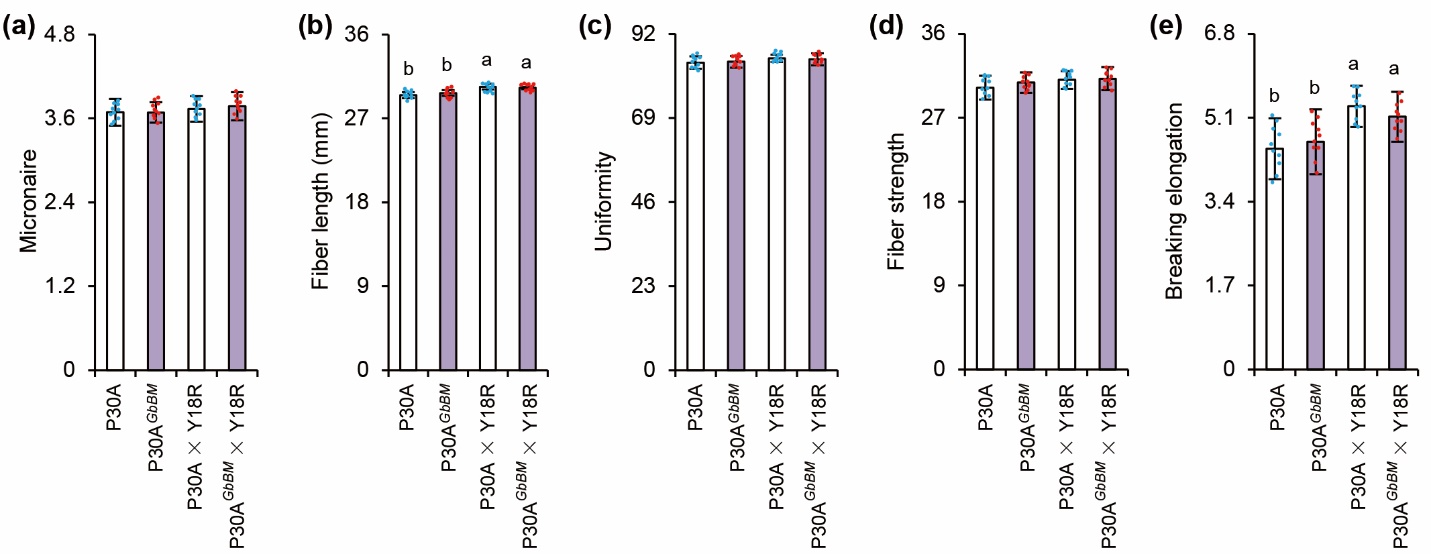
**

**Figure S9 Fiber quality traits of P30A and P30A*^GbBM^* after crossing with Y18R by honeybee mediated pollination.** Micronaire **(a)**, fiber length **(b)**, uniformity **(c)**, fiber strength **(d)**, and breaking elongation **(e)** of P30A and P30A*^GbBM^*. The data were analyzed by one-way ANOVA followed by an LSD test. Different letters above the bars indicate a significant difference at *P* < 0.05.

**Table S1** Genetic analysis using F_2_ population derived with *G. hirsutum* line P30A and *G. barbadense* line HaiR. The observed ratios were tested for deviation from the expected values with a Chi-Square test for goodness-of-fit (*P* < 0.05).

|  | Number of plants with purple  spot at the base of flower petals | Number of plants without purple  spot at the base of flower petals |
| --- | --- | --- |
| Expected phenotypic ratio | 0.75 | 0.25 |
| Observed ratios | 4255 | 1285 |
| *p*-value | 0.0019 | |

**Table S2** Primers used in this study.

|  | Forward Primers | Reverse Primers |
| --- | --- | --- |
| **Primers for map-based cloning** | | |
| CMB1 | AGTCCAACTAGCGAGCTTTAAGT | TGTCAGATCGACTTGAATCTAAGGT |
| CMB2 | GCATTTTGAAGTAGCGAAAATCTCTG | TTTCAAGTAAAGCACCACTCGA |
| CMB3 | GGGTTAATGGGTTTTTAGCGGG | ACAATTGCACCCTTAGACGAGT |
| CMB4 | TGAAGTAGCGAAAATCTCTGTAACT | TCAAGTAAAGCACCACTCGATTAT |
| CMB5 | GGGCTGTGCTATTGGGTTTAAG | AGAATTCGCAGTATAGGTGTTCT |
| CMB6 | ACAACCATGTCAGCCCTGTATT | TTTCGACTCACTTCACCAAGCA |
| CMB7 | TAATTGAACCTGACCGAGCTCG | TATTCGCCTCCATAACCTCCTC |
| CM7817 | CCATCAACCGAGGGGAAATAGT | GGGAATGAGAAAGGGACATCAAATT |
| CM7819 | ACTAAACTACAAGCATCGAAAATGG | TCCTTCATGGTCGTCTTCAATATAG |
| CM7290 | TCGTTGTGCTCTTTAGATAGTATCT | TCCAATCAGCTTACCTATTTGAACT |
| CM7296 | AGTCATGCTCCAGTTGATACGG | GGGCCTCTTCTAAAGAAAGGCT |
| **Primers for sequencing of candidate ORFs** | | |
| ORF1 | ATGGCACTAGCCAAGGACCCTA | TTAGCTTGAATTTATATCTTTC |
| ORF2 | ATGGATAAGAATGTGGGAATTC | TTAGTGCTTATTGACTACCTCA |
| ORF3 | ATGGATGATGAAGAGAAGGCTA | CTAGAATATTTTAGCAACATCC |
| ORF4 | ATGGCTGCTCTTTACTCAACTT | TTAGGCCACGGTTTTGACGAGA |
| ORF5 | ATGGCGAGTTTTTTAGGTTCAA | TTAATTTGAAGCATTGGTCATC |
| ORF6 | ATGGAAGCTCCATCTTTAAGCG | CTATGGGTTGAGCATATTCCAA |
| ORF7 | ATGGAAGGCTCATCTTTAAGAG | CTATGGGTTGAACACATTCCAC |
| GbBM_CDS_ | ATGGAGGGCTCATATTTAAG | CTATGGGTTAAACACATTCCACA |
| GhBM_CDS_ | ATGGAGGGCTCATATTTAAG | CTATGGGTTAAACACATTCCACA |
| GbBM_Promoter_ | GCTATTTGTATTCTAGTTTGC | ATAGAAGCTGTTATAACTAGCT |
| GhBM_promoter_ | GCTATTTGTATTCTAGTTTGC | ATAGAAGCTGTTATAACTAGCT |
| **Primers for cloning of *GbBM* gene** | | |
| GbBM | ATGGAGGGCTCATATTTAAG | CTATGGGTTAAACACATTCC |
| GhBM | ATGGAGGGCTCATATTTAAG | CTATGGGTTAAACACATTCC |
| **Primers for VIGS** | | |
| VIGSprimers | TAAGGTTACCGAATTTGCTGCTGATGAAGTTGACC | GCTCGGTACCGGATCATCCATCGTGGTTGTCACTGC |
| **Primers used for vectors construction** | | |
| GbBM_CDS_-eGFP | GGGTACCCGGGGATCATGGAGGGCTCATATTTAAGAGT | CGACTCTAGAGGATCTGGGTTAAACACATTCCACAGT |
| GbBM_Promoter_-LUC | ACTAGTAGAAGGCCAAACAACGACGA | GGATCCTCCGCAGAAAAGTTCCTCGT |
| GhBM_Promoter_-LUC | ACTAGTAGAAGGCCAAACAACGACGA | GGATCCTCCGCAGAAAAGTTCCTCGT |
| GbBM_CDS_-LUC | AGATCTATGGAGGGCTCATATTTAAG | GGATCCCTATGGGTTAAACACATTCC |
| GhBM_CDS_-LUC | AGATCTATGGAGGGCTCATCTTTAAG | GGATCCCTATGGGTTAAACACATTCC |
| GbBM_CDS_-Y1H | TGCCTCTCCCGAATTCATGGAAGCTCCATCTTTAAGCG | CGAGTCGGCCCTCGAGTCACACCCCTAATGCTTCATTGA |
| Gb4CL_Promoter_ | TTGAATTCGAGCTCGGTACCGTGCAAATGCAATCCCAATC | TCGACAGATCCCCGGGTACCATTTGCAATTCAGCCCTCTT |
| GbCHS_Promoter_ | TTGAATTCGAGCTCGGTACCAAATAGCCCCGATCCGATAC | TCGACAGATCCCCGGGTACCGAATGGGTAGGTTGGATGGA |
| GbCHI_Promoter_ | TTGAATTCGAGCTCGGTACCTGGCACGTTAATTCAAATGG | TCGACAGATCCCCGGGTACCCCCTGTTTGTGGTGGTAGGT |
| GbF3H_Promoter_ | TTGAATTCGAGCTCGGTACCCCGATTCCCCTGTTGTTGTA | TCGACAGATCCCCGGGTACCCCGACTTGTCATTTTCTTCG |
| GbFLS_Promoter_ | TTGAATTCGAGCTCGGTACCGAGTTCGTTTGTCGTTGTTG | TCGACAGATCCCCGGGTACCCTCCTGCTTTGTGTTCCCTA |
| GbDFR_Promoter_ | TTGAATTCGAGCTCGGTACCGATTGGAGCCTGTCGATGTT | TCGACAGATCCCCGGGTACCACGACCGGTAGTTGGTTGAG |
| GbANS_Promoter_ | TTGAATTCGAGCTCGGTACCACCAACGCATGTGAAGCTCT | TCGACAGATCCCCGGGTACCGTGGGATTTTTATAGGGCAGTG |
| GbRT_Promoter_ | TTGAATTCGAGCTCGGTACCAACCATGTCCCATCCACCTA | TCGACAGATCCCCGGGTACCTGTTTGCTCATGTTGGGCTA |
| GbUFGT_Promoter_ | TTGAATTCGAGCTCGGTACCTTTGCCGTCTTGACAGACAC | TCGACAGATCCCCGGGTACCATGGGTAAGGGCAGGAAACT |
| **Primers for ChIP-PCR** | | |
| GbCHS_Promoter_-P1 | GCATCATGCCACCACTCTAA | TGTTGCCAATACAAAGATCTGG |
| GbCHS_Promoter_-P2 | GATGGCAAATGTTCCTGCTT | AGGCCACCATTTTCCTTTCT |
| GbCHS_Promoter_-P3 | TTTATTTTGGGCCAGGTTTG | GCCCCATCCCTGTCTATTTC |
| GbCHS_Promoter_-P4 | GCCCCCACCTCTAACCTTTA | GTCAGTCATGTGGTCGCTGT |
| GbFLS_Promoter_-P1 | CCATACATTGGATGAAGAAGCA | CCCCCGCATTCTTTTATACC |
| GbFLS_Promoter_-P2 | CGAGGATGATGATGACGATG | CGCAGAGAGGAAAACCTGAA |
| GbFLS_Promoter_-P3 | TTGCTATTGAGCTGACGTGTG | CGAATCAATAAAACCATCAATTC |
| GbFLSS_Promoter_-P4 | AGGTTTTCATGGCAGTCTGA | TCTCAGGCAGGAGGACATTT |
| GbDFR_Promoter_-P1 | TTCAAGTCGGTGCTTACTGC | TCGGTCATGTCCCTGTATGA |
| GbDFR_Promoter_-P2 | CACATCGAGACTGTACTTTG | CGATGGTCTTAGAGGAGTTG |
| GbDFR_Promoter_-P3 | AGTGGGAGGGGAAAGTGTT | CTCACCCTAATCAAAGCTAC |
| GbDFR_Promoter_-P4 | TGAAGAAGCAGCACCTGAA | ATATGGCGCAGTCCTTCAAT |
| GbANS_Promoter_-P1 | TGTCATTTTAAAGTTTGATGAG | AAAAGAAGATCCGGGAGGAA |
| GbANS_Promoter_-P2 | TTCCTCCCGGATCTTCTTTT | GGAAGCAAATATGAAGCCAATC |
| GbANS_Promoter_-P3 | TACTATCGTCAAAAATATGCA | CCATTCTACGCAAAGCCATT |
| GbANS_Promoter_-P4 | AATGGCTTTGCGTAGAATGG | CAGGAACATGCCGGTTCTAA |
| **Primers for qRT-PCR** | | |
| GbACT1 | GAAGGCATTCCACCTGACCAAC | CTTGACCTTCTTCTTCTTGTGCTTG |
| GhACT1 | GAAGGCATTCCACCTGACCAAC | CTTGACCTTCTTCTTCTTGTGCTTG |
| GbBM | TGCTGCTGATGAAGTTGACC | TGATAAGATCTGAGGCCGAGG |
| GhBM | TGCTGCTGATGAAGTTGACC | TGATAAGATCTGAGGCCGAGG |
| ORF1 | TCGACGTGAGAGGATCAGTG | ATCAGGTGCTTTCCCTCCTT |
| ORF2 | AAGTTGAAACCGAGGAAGCA | CGATTTGTTTGGGGTCAATC |
| ORF3 | AATCAGTGGGACTCGGACAC | TCAACACGAAGCAAAGCAAC |
| ORF4 | AGGCTAACAACGATCGCATT | CGTCCCCATCAGTATCCATT |
| ORF5 | TTTCGGGGAAATGCTGATAG | CAACCTTCCCGATAAAAGCA |
| ORF6 | TAAAGACGGGAATGGCTACG | AATCATTCTCCGGCAATCAC |
| ORF7 | TCGCCTGCATAACCTTCTTG | TTGCTGGTGTTGATGCTGC |
| Gbar_A01G021170 | ACAACGGTCTCCCATCAAAC | GACTGCTTCGGCTGTTTTTC |
| Gbar_A06G007820 | TCATGAGCTCAAGCACCATC | GTCCAAGCCACTGAGGAGAG |
| Gbar_A09G015990 | GGAAATGCTGCAATCCCTAA | CCTTGGCACATAGCTGTGAA |
| Gbar_D05G000570 | GTCGCTTCCACAAAACCAAT | TTGGGATGGGCAATTAACAT |
| Gbar_D10G005150 | GGCTGGTTACATACCGGAGA | TCACCACAAATGCAACAGGT |
| Gbar_A10G005330 | ACATCGGCCTACTGTTGGAG | ATTTGGCACCAGGAAACTTG |
| Gbar_A09G026740 | ACCCTCTGATACGCACTTGG | AATTGCCCCATCTGAATCTG |
| Gbar_D02G003130 | ATCAGGTTGAGGCGAAGCTA | CGTGGTTCCAAGTCCATCTT |
| Gbar_D10G015020 | GACAGTCTAGTGGGCCAAGC | TTCACGAAGGTGACCATCAA |
| Gbar_A09G000080 | GCGCTGCAGCTGTCATAATA | GATGGAGTTCCAGTCCCAGA |
| Gbar_D13G002040 | AAACTTCCCACCTGGCTCTT | TCTTGATGCCAAGCTTTCCT |
| Gbar_D11G019050 | GCAATGGGATTGGAGAAAGA | TTGGAGCAACAGCGTGATAG |
| Gbar_A06G000690 | CCTGGAAGTTTGCAAAGGAG | TGCAGAGATCATCCAAGTGC |
| Gbar_D04G002320 | TTGGGGTAACATCCCACACT | CTGTTGTTCGATGCAGCACT |
| Gbar_A05G039600 | TGAAGGGTATGGAAGCAAGG | TGCGCATACTCTTCGTTCAC |
| Gbar_D07G007140 | CAACAATCGCCTGACAAGAA | GCTGGGAAGTAACGAAGTGC |
| Gbar_A12G023810 | GCGGGCTAGAGAAGGAAGTT | CATTTCGCGGTAATCCACTT |
| Gbar_D08G010280 | AAGGAGTTGGAGCCCTTGAT | TCCATGATTGATTGCCTGAA |
| Gbar_D09G017370 | TGCCCTGAAATCTGAAATCC | CTCAAAGAAGTCACGCCACA |
| Gbar_A02G003060 | GTCCGCCGATAGAAAAATCA | TTGCTGAATTCCAACCACAA |
| Gbar_D08G010890 | CCTTCATCTGGTGTGGTGAA | CAGTCGCATATTTTCCAGCA |
| Gbar_D07G008670 | GCCGGAAAAGCTGTAGACTG | CGTTTGCTGTTCTTCCTGGT |
| Gbar_A07G008300 | TCGCCTGCATAACCTTCTTG | TTGCTGGTGTTGATGCTGC |
| Gbar_D07G008660 | AAAGGTGCATGGACTGAAGA | AGGAGGTTATGGAGGCGAAT |
| Gbar_A07G008290 | TAAAGACGGGAATGGCTACG | AATCATTCTCCGGCAATCAC |
| Gbar_A13G011420 | GAGGGAGTTTTGCAGCAGAG | AGGAGATACATGAGCTGGGC |
| Gbar_D11G026500 | GCTCCTCGGAAACAAATGGT | GTGGCTGGGGTTTTAGGGTT |
| Gbar_A11G025580 | CGCCCAAACATCAAAAGAGGA | GTGGCTGGGGTTTTAGGGTT |
| Gbar_D13G011200 | GGTTGCTTGGACGGAAGAAG | CGTCTGGGTTTCATTGAGGC |

**Table S3** Candidate ORFs between the mapping markers CMB5 and CMB7. The data were extracted from CottonFGD (https://cottonfgd.org/).

|  | *Gene ID* | *Gene Description* | *Genomic DNA Length (bp)* | *CDS Length (bp)* | *Protein Length (aa)* |
| --- | --- | --- | --- | --- | --- |
| ORF1 | *Gbar_A07G008240* | Transcription factor bHLH086 | 2326 | 885 | 271 |
| ORF2 | *Gbar_A07G008250* | Hydroxymethylglutaryl-CoA synthase | 3861 | 414 | 137 |
| ORF3 | *Gbar_A07G008260* | Probable calcium-binding protein CML28 | 249 | 249 | 82 |
| ORF4 | *Gbar_A07G008270* | Probable fructokinase-6 | 3864 | 1167 | 358 |
| ORF5 | *Gbar_A07G008280* | Probable calcium-  binding protein CML23 | 678 | 465 | 154 |
| ORF6 | *Gbar_A07G008290* | Transcription factor MYB113 | 2979 | 648 | 215 |
| ORF7 | *Gbar_A07G008300* | Transcription factor MYB113 | 6150 | 744 | 245 |
| ORF8 | *Gbar_A07G008330* | Transcription factor MYB113 | 5043 | 750 | 249 |

**Table S4** Haplotypes of Beauty mark between *G. hirsutum* line P30B and *G. barbadense* line HaiR. V1-8 indicates the variants, and their positions relative to ATG are shown in the table.

| **Haplotype** | **P30B** | **HaiR** |
| --- | --- | --- |
| V1. -211 | T | C |
| V2. -411 | G | A |
| V3. -912 | GAAA | ---- |
| V4. -980 | T | A |
| V5. -1067 | -- | TT |
| V6. -1203 | --- | ATT |
| V7. -1208 | T | A |
| V8. -1671 | T | G |
